# Healthy Dietary Patterns and Risk of Sarcopenia in Adults Aged > 50 Years: A Systematic Review and Meta-Analysis Considering EWGSOP1 and EWGSOP2 Criteria

**DOI:** 10.3390/nu17172764

**Published:** 2025-08-26

**Authors:** Roxana E. Ruiz-Valenzuela, Reyes Artacho, María Dolores Ruiz-López, Esther Molina-Montes

**Affiliations:** 1 Department of Health Sciences, Universidad Iberoamericana Tijuana, Tijuana 22500, Mexico; roxana.ruiz@tijuana.ibero.mx; 2 Department of Nutrition and Food Science, Universidad de Granada, 18071 Granada, Spain; mdruiz@ugr.es (M.D.R.-L.); memolina@ugr.es (E.M.-M.); 3 Institute of Nutrition and Food Technology (INTA) Jose Mataix, Universidad de Granada, 18071 Granada, Spain; 4 CIBER of Epidemiology and Public Health (CIBERESP), 28029 Madrid, Spain; 5 Instituto de Investigación Biosanitaria ibs.GRANADA, 18012 Granada, Spain

**Keywords:** sarcopenia, dietary patterns, EWGSOP1, EWGSOP2, meta-analysis

## Abstract

Background/Objectives: Sarcopenia is characterized by the progressive loss of skeletal muscle mass and function. Little is known about the dietary patterns and sarcopenia association. The aim of this systematic review and meta-analysis was to evaluate the association between dietary patterns and the risk of sarcopenia in adults over 50 years old, using the European Working Group on Sarcopenia in Older People, EWGSOP1 and EWGSOP2 criteria. Methods: This review followed PRISMA guidelines and was registered in PROSPERO (CRD42024423323). A systematic search was conducted in PubMed, Web of Science, and Cochrane Library (July 2024–February 2025). Observational studies in adults aged 50–85 years assessing a priori or a posteriori dietary patterns were included. Odds ratios (ORs) and 95% confidence intervals (CIs) were extracted. A random-effects model was used for meta-analysis. I^2^ and meta-regression was performed to explore heterogeneity sources. Methodological quality was assessed with the Joanna Briggs Institute (JBI) checklist. Results: Eleven studies were included (*n* = 257–3432). The pooled analysis showed a significant 24% risk reduction in sarcopenia with healthy dietary patterns (OR = 0.76, 95% CI: 0.63–0.92, I^2^ = 56.2). Unhealthy dietary patterns did not show a significant association (OR = 1.04, 95% CI: 0.66–1.63). Mediterranean (MD) pattern yielded the strongest effect (OR = 0.62, 95% CI: 0.40–0.95). Meta-regression analyses did not identify significant variables. Conclusions: Healthy dietary patterns, particularly the MD, are associated with a lower risk of sarcopenia in adults over 50 years old and represent a promising nutritional strategy for sarcopenia prevention.

## 1. Introduction

Sarcopenia is a musculoskeletal disease characterized by the progressive loss of skeletal muscle mass and function, often associated with aging and leading to adverse outcomes such as frailty, falls, and poorer health [1,2]. Globally, sarcopenia affects an estimated 10–16% of older adults, although its prevalence varies significantly depending on the diagnostic criteria used [1,3,4] and across different regions [5]. Several scientific organizations have developed consensus criteria for sarcopenia [6,7,8].

The European Working Group on Sarcopenia in Older People (EWGSOP) originally established a consensus definition in 2010 (EWGSOP1), in which sarcopenia was diagnosed by the presence of low muscle mass in conjunction with either low muscle strength or low physical performance. This framework placed muscle mass at the core of the diagnosis, with muscle strength and physical performance as supportive criteria. EWGSOP1 distinguished between the following: pre-sarcopenia (low muscle mass only), sarcopenia (low muscle mass with low strength or poor physical performance), and severe sarcopenia (low muscle mass, low strength, and poor physical performance) [6]. In 2019, EWGSOP published a revised consensus (EWGSOP2) that shifted the diagnostic focus to low muscle strength as the principal criterion, based on evidence that muscle strength declines faster and is a stronger predictor of adverse outcomes than muscle mass [2]. EWGSOP2 distinguished probable sarcopenia (low muscle strength), confirmed sarcopenia (low muscle strength and low muscle mass), and severe sarcopenia (low muscle strength, low muscle mass, and poor physical performance).

In other regions, such as Asia, the Asian Working Group for Sarcopenia (AWGS and AWGS2019) [9,10] established criteria with region-specific cut-off points for muscle mass and strength, adapted to the anthropometric and body composition characteristics of Asian populations. At the global level, EWGSOP and AWGS remain the most widely adopted definitions in prevalence studies [10,11,12]. Current evidence shows that applying different diagnostic criteria to the same population can yield marked variations in estimated sarcopenia prevalence (Asian populations: 4–16.1%; European populations: 7–16.8%), highlighting the need for definitions tailored to specific ethnic and geographic contexts [5,10,13]. Therefore, the use of EWGSOP criteria facilitates comparability across studies conducted in Western populations.

Sarcopenia involves complex physiological and pathological interactions such as neuromuscular junction degeneration [14], mitochondrial dysfunction [15], hormonal alterations, and chronic inflammation [16]. Modifiable lifestyle factors such as physical inactivity and poor nutrition have been associated with this disease [17,18]. Among these factors, diet represents a central lifestyle factor with direct influence on muscle metabolism and sarcopenia risk [18]. Certain dietary factors may prevent or at least delay the onset of sarcopenia by improving some of the aforementioned pathological processes. For instance, protein and essential amino acids, particularly leucine, are necessary for stimulating muscle protein synthesis and preserving muscle mass, especially in older adults with increased anabolic resistance [19,20]. Higher protein intake is a key regulator of bone formation and anabolic responsiveness [21]. In addition, vitamins D, C, E, and B-complex, as well as minerals such as calcium, magnesium, and selenium, support muscle function by promoting protein turnover, combating oxidative stress, and modulating inflammation [22,23]. Omega-3 fatty acids have also been associated with improvements in muscle mass and strength, likely through anti-inflammatory and membrane-stabilizing effects [24].

However, while initial research emphasized the effects of individual nutrients, increasing evidence highlights the importance of whole-diet approaches in understanding musculoskeletal health [25,26,27]. Compared to single-nutrient analyses, dietary pattern approaches may offer a more integrated and realistic framework for evaluating diet–muscle health relationships [27]. Two methodological approaches, a priori and a posteriori dietary pattern analyses, have been employed to study the relationship between diet and muscle health. A priori approaches in dietary pattern analyses use predefined dietary indices (e.g., the Mediterranean Diet Score (MDS), the Dietary Approaches to Stop Hypertension (DASH Score), or the Alternative Healthy Eating Index (AHEI)), whereas a posteriori approaches identify dietary patterns through statistical techniques such as principal component analysis and cluster analysis [28]. Within this framework, recent studies applying both approaches have suggested that high-quality diets could be associated with muscle health [26,27,29]. For instance, the Mediterranean Diet (MD), with its anti-inflammatory properties attributed to its high content of antioxidants and healthy fats, such as extra virgin olive oil, may contribute to the preservation of muscle mass and function. In fact, higher adherence to the MD acts as a protective factor against functional disability and reduced risk of developing sarcopenia in some studies [25,30,31].

Nevertheless, while the MD appears beneficial, previous reviews examining a wider range of healthy dietary patterns have yielded mixed and inconclusive results. Indeed, a meta-analysis of four prospective cohort studies concluded that evidence linking dietary patterns to sarcopenia in older adults is still limited and inconsistent [32]. Similarly, another meta-analysis found associations with gait speed, but not another sarcopenia-related outcomes [33]. These findings are further complicated by considerable heterogeneity in dietary exposures, assessment methods, and outcome definitions across studies [32,33,34,35]. In particular, variation in sarcopenia definitions has led to the inclusion of studies using divergent diagnostic criteria, which could potentially dilute observed associations and limit clinical interpretability or comparability between the studies addressing this association [36].

In light of this methodological heterogeneity and the lack of consensus in previous reviews, the present systematic review and meta-analysis aims to assess the association between healthy and unhealthy dietary patterns and sarcopenia risk by focusing on studies that applied diagnostic criteria established by EWGSOP1 or EWGSOP2.

## 2. Materials and Methods

### 2.1. Study Design

Our systematic review protocol was registered in the International Prospective Register of Systematic Reviews (PROSPERO) under registration number CRD42024423323. The study adhered to the Preferred Reporting Items for Systematic Reviews and Meta-Analyses (PRISMA) guidelines, ensuring transparency and rigor in the selection, extraction, and synthesis of data [37]. The PRISMA checklist is available in Supplementary Table S1.

### 2.2. Eligibility Criteria

The inclusion criteria were (a) observational studies and (b) studies that assessed sarcopenia based on the diagnostic algorithm established by either EWGSOP1 or EWGSOP2. However, for the purposes of this review, only the prevalence of sarcopenia (no severe sarcopenia) was considered. Additionally, only studies conducted in human populations and published in English were included.

Exclusion criteria were (a) studies that assessed only one of the sarcopenia components, (b) studies involving participants under 50 years of age, and (c) studies where the primary outcomes were specific diseases unrelated to sarcopenia.

A comprehensive search of the literature was conducted in three major electronic databases: PubMed, Web of Science, and Cochrane Library, from July 2024 to February 2025. The search strategy incorporated Medical Subject Headings (MeSH) terms and keywords related to dietary patterns, sarcopenia, muscle strength/mass, and physical function. The research project can be summarized with the following PICOs format:

P (Population): Non-institutionalized adults over 50 years diagnosed with sarcopenia either with EWGSOP1 (2010) or EWGSOP2 (2019). I (Intervention/Predictors): Dietary patterns, including a priori and a posteriori derived patterns. C (Comparator): Individuals with low adherence vs. high adherence to the identified dietary patterns or indices. O (Outcome): Sarcopenia risk, diagnosed using the criteria established by EWGSOP1 or EWGSOP2. S (Study design): Observational studies (cross-sectional or longitudinal).

### 2.3. Search Strategy

The search strategies implemented in each database were as follows:

Pubmed: (((((“Dietary Patterns”[Mesh]) OR (“Dietary Score”[Title/Abstract]) OR (“Diet* Quality”[Title/Abstract]) OR (“Diet* Index*”[Title/Abstract]) OR (“A Priori Diet*”[Title/Abstract]) OR (“A Posteriori Diet*”[Title/Abstract]))) AND ((“Sarcopenia”[Mesh]) OR (“Physical Functional Performance”[Mesh]) OR (“Muscle, Skeletal”[Mesh]) OR (“Muscle Strength”[Title/Abstract]) OR (“Muscle Mass”[Title/Abstract]) OR (“Muscle Function”[Title/Abstract]) OR (“EWGSOP”[Title/Abstract] OR “EWGSOP2”[Title/Abstract]))))

Web of Science: ((((((TS=(“Dietary Pattern”)) OR TS=(“Dietary Score”)) OR TS=(“Diet* Quality”)) OR TS=(“Diet* Index*”)) OR TS=(“A Priori Diet*”)) OR TS=(“A Posteriori diet*”)) NOT (SILOID==(“PPRN”)) AND ((((((TS=(Sarcopenia)) OR TS=(“Physical Functional Performance”)) OR TS=(“Hand Strength”)) OR TS=(“Muscle Strength”)) OR TS=(“Muscle, Skeletal”)) OR TS=(“Geriatric Assessment”)) OR TS=(“EWGSOP”) and Preprint Citation Index (Exclude—Database)

Cochrane: #1 “Dietary Patterns” OR “Dietary Score” OR “Diet Quality” OR “Diet index” OR “A Priori Diet” OR “A Posteriori Diet” OR “Dietary Score” OR “Diet Quality Index #2 “Sarcopenia” OR “Grip Strength” OR “Handgrip” OR “Physical Performance” OR “Muscle Strength” OR “Gait Speed” OR “EWGSOP” #3 “Aged” OR “Aging” OR “Older Adults” #4 #1 AND #2 AND #3

### 2.4. Study Selection and Data Extraction

Two independent reviewers, RERV and RA, conducted a comprehensive screening process, beginning with the evaluation of titles and abstracts, followed by full-text reviews to determine study eligibility. Any discrepancies were resolved through discussion or consultation with a third reviewer, EMM.

Data extraction included study characteristics such as author, publication year, country, study name, sample size, sex distribution (%), mean age, dietary intake assessment, dietary pattern type, and dietary pattern name. In addition, data on sarcopenia prevalence; diagnostic methods; specific cutoff points for muscle mass, muscle strength, and physical performance; and statistical outcomes such as odds ratios (ORs), 95% confidence intervals (CIs), and *p*-values and adjusted covariates were extracted. For prospective studies, only baseline, i.e., cross-sectional, data were extracted.

### 2.5. Quality Assessment

The methodological quality of the included studies was assessed using the The Joanna Briggs Institute (JBI) checklist, applying the 8-item version for cross-sectional studies [38]. The checklist evaluates key methodological domains, including sample selection, measurement validity, confounding control, and statistical analysis. Each item was rated as 1 (criterion met) or 0 (criterion not met), and a total score was calculated for each study. Although study quality was assessed based on the total JBI score, studies were classified as high quality (≥6 points) or low quality (<6 points). Data extraction and quality assessment were independently performed by the two aforementioned reviewers, and inconsistencies were solved by consensus or involving the third reviewer.

### 2.6. Data Synthesis

The meta-analysis was conducted to pool the ORs and their 95% CIs for the association between dietary patterns and sarcopenia risk. Dietary patterns were classified as healthy or unhealthy. Healthy dietary patterns emphasized nutrient-dense and anti-inflammatory foods (fruits, vegetables, whole grains, unsaturated fats, nuts, legumes), in accordance with WHO [39], FAO/WHO [40] and well established dietary quality indices. Unhealthy dietary patterns featured higher intakes of pro-inflammatory or metabolically adverse foods (processed meats, refined grains, sugar-sweetened beverages, sweets/desserts, high-sodium foods, and saturated/trans fats) [41,42]. For each study, the classification was based on the original authors’ description of the dietary pattern and the predominant food groups contributing to it.

The first analysis considered the studies assessing associations for a priori healthy dietary patterns: Alternative Healthy Eating Index 2010 (AHEI-2010) [43], Australian Dietary Guideline Index (DGI) [44], Baltic Sea Diet (BSD) [45], Dietary Approaches to Stop Hypertension (DASH) [46], Dietary Inflammatory Index (DII) [47], Modified Healthy Diet Index (mHDI), Modified Mediterranean Diet Score (mMDS) [48], Mediterranean Diet Score (MDS), and Nutrient-Risk Variable (NRV) [44]. As for the a posteriori patterns, the healthy patterns were as follows: anti-inflammatory pattern (Anti), carbohydrate vitamin pattern (Carbo-vit) [49], Low Red Meat vs. Low Butter [50], Vegetable & Fruit [51], Mediterranean pattern [52,53], and protein vitamin pattern (Pro-vit) [49]. To maintain consistency in the interpretation of results, the odds of DII was inverted, as this dietary pattern represents a harmful effect with increasing levels of adherence [47]. Similarly, in another study [52], the scoring was also inverted to reflect a comparison between high vs. low adherence to the MD, thereby ensuring alignment with the direction of the effect across all studies. This approach allowed for direct comparison between healthy dietary patterns within the same cohort, enhancing the robustness of the meta-analysis and minimizing potential interpretative bias by evaluating both beneficial and detrimental dietary exposures under a unified methodological framework.

Secondly, the analyses focused on unhealthy dietary patterns identified both a priori and a posteriori. The a priori pattern was represented by the DII [47], while the a posteriori patterns included the Western and Mixed dietary patterns [53], Milk & Cereal, Bread & Cheese, Meat & Egg [40], and Traditional British vs. Low Butter patterns [50].

Random-effects models were used as the primary approach to minimize potential heterogeneity driven by differing study populations, dietary assessment methods, and sarcopenia definitions. Statistical heterogeneity was assessed using the I^2^ statistic, with values of 25%, 50%, and 75% considered low, moderate, and high heterogeneity, respectively. Significant heterogeneity was considered when I^2^ > 50%. A meta-regression was conducted to identify potential sources of heterogeneity by assessing the influence of study-level covariates such as age, sex, geographic region, quality of studies, dietary assessment method, diagnostic criteria of sarcopenia, and dietary pattern type. The geographic region was characterized by continent: Oceania [44], Middle east [43,46,47,49,53], and Europe [45,48,50,51,52].

In fact, this analysis aimed to explore whether these factors could explain the variability in effect sizes observed across studies, particularly given the differences in diagnostic criteria (EWGSOP1 vs. EWGSOP2), dietary pattern classification (a priori or a posteriori), and population characteristics. A separate sensitivity analysis was conducted excluding studies classified as low-quality (<6 points in the JBI Checklist) to evaluate the robustness of the findings.

Risk of bias was assessed using visual inspection of funnel plots and accompanying Egger’s tests. Statistical analyses were performed using Stata version 18.0 (Stata Corp, College Station, TX, USA). A *p*-value < 0.05 was considered statistically significant for all analyses.

## 3. Results

### 3.1. Study Selection

**Figure 1:**
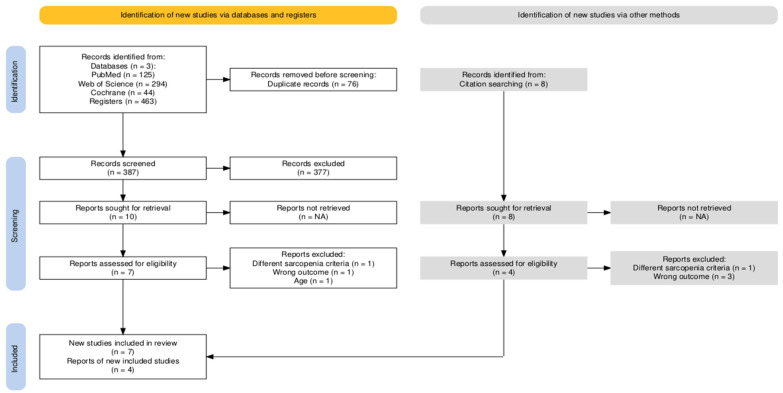
PRISMA flow diagram used to record the selection process in the current study.

The screening process is outlined in Figure 1. A total of 463 publications were initially identified through database searches (PubMed: n = 125, Web of Science: n = 294, Cochrane: n = 44). After the removal of 76 duplicate records and the exclusion of 377 studies that did not meet the eligibility criteria during the title and abstract screening, eleven full-text articles were assessed for eligibility. Following the full-text evaluation, four studies were excluded, while four additional studies were included through citation searching, resulting in a final sample of eleven eligible studies for the meta-analysis (Figure 1).

### 3.2. Study Characteristics

Table 1 presents the characteristics of the included studies with reference to country, sex, age, dietary assessment method, and types of dietary patterns analyzed. The selected studies were conducted across six different countries, including Iran [43,46,47,49,53], Finland [45], Sweden [48,51], United Kingdom [50], Australia [44], and Italy [52]. Sample sizes ranged from 257 to 3432 participants. Regarding the sex distribution, three studies exclusively included men [44,48,51], one study focused only on women [45], and seven studies included a mixed population [43,46,47,49,50,52,53]. The age of the participants ranged from 50 [41] to 85 years [39].

Dietary intake was assessed using a variety of validated instruments, including a 3-day food record [45], a 7-day diet record [48,51], Food Frequency Questionnaires (FFQs) [43,46,47,49,53], a diet history questionnaire [44], a 24 h multiple-pass recall [50], and a combination of FFQ and 24 h recall methods [52].

#### 3.2.1. Dietary Patterns

A priori dietary indices were applied in seven studies, including the AHEI-2010 [43], Australian DGI [44], BSD [45], DASH Diet Score [46], DII [47], mHDI and mMDS [48], as well as MDS and NRV [44]. A posteriori approaches were used in five studies, identifying patterns such as Mediterranean, Western, and Mixed [53], Low Red Meat vs. Low Butter [50], four specific dietary patterns (Milk & Cereal, Vegetable & Fruit, Bread & Cheese and Meat & Egg) [51], and the Anti, Carbo-vit, and Pro-vit pattern [49]. Eight of the included studies were cross-sectional in design [44,46,47,49,50,52,53]. Additionally, three cohort studies were included by analyzing data from their baseline assessments [48,50,51], allowing for their integration into the cross-sectional analysis. Supplementary Table S2 shows the characteristics of dietary patterns according to whether they are classified as healthy or unhealthy.

#### 3.2.2. Sarcopenia Diagnosis

Table 2 presents the diagnostic criteria, component measurement and cutoff points employed to assess sarcopenia. Sarcopenia was diagnosed using EWGSOP1 in ten studies [43,44,45,46,47,48,49,50,51,53] while EWGSOP2 was applied in five studies, either alone [41] or in combination with EWGSOP1 [43,44,46,51]. The reported prevalence of sarcopenia varied depending on the diagnostic criteria used and the population studied, ranging from 9% to 23.2%. The highest prevalence (23.2%) was observed in the OSPTRE-EPS Study (Finland; n = 3432) [45], whereas the lowest prevalence 9% (n= 170) was reported in Mazza et al., 2024 [52].

Muscle mass was most commonly assessed using Dual-energy X-ray Absorptiometry (DXA) [43,44,45,46,47,48,49,51,53], while Bioelectrical Impedance Analysis (BIA) was employed in two studies only [50,52]. Cutoff points for appendicular Skeletal Muscle Mass (ASM) [43,44,45,46,47,48,49,51,53], Skeletal Muscle Index (SMI) [50], or Relative Skeletal Muscle Index (RSMI) [34] were adapted by sex and standard reference values in all studies.

Handgrip strength (HGS) was the standard method for assessing muscle strength in all studies. Seven studies applied the cut-off values recommended by EWGSOP1 or EWGSOP2 [44,46,48,49,50,51,52] and other applied different cutoff points [43,45,47,53].

Physical performance was assessed primarily through gait speed over 4 to 10 m [43,44,45,46,47,48,49,51,52,53]. Additionally, one study included the 5-time chair stand test [51], and another used the Timed Up and Go test without gait speed [50].

Furthermore, Table 2 presents findings on the association between dietary patterns and sarcopenia risk. Among the a priori indices, the inverse DII [47] showed the strongest protective association (OR = 0.46, 95% CI: 0.22–0.99) in multivariate-adjusted models. In contrast, the mHDI and mMDS [48] showed no significant associations under EWGSOP1 criteria (mHDI: OR = 0.47, 95% CI: 0.17–1.28; mMDS: OR = 0.33, 95% CI: 0.09–1.23). Similarly, another study reported null associations for both the MDS (OR = 1.05, 95% CI: 0.44–1.66) and the BSD (OR = 0.93, 95% CI: 0.38–1.48) [34]. Three other studies evaluating the DGI, MDS, NRS, DASH, and AHEI-2010 indices found no significant associations with sarcopenia, regardless of whether EWGSOP1 or EWGSOP2 criteria were applied [43,44,46].

Regarding a posteriori approaches, MD pattern was associated with reduced sarcopenia risk (OR = 0.40, 95% CI: 0.17–0.97) [53], while Western and Mixed dietary patterns did not show significant associations, despite models being adjusted for lifestyle and clinical factors. Likewise, another MD pattern [52] was significantly associated with lower risk of sarcopenia under EWGSOP2 criteria (OR = 0.10, 95% CI: 0.015–0.71), although specific adjustment variables were not reported. Similarly, Anti pattern [47] reported a strong protective association with sarcopenia risk (OR = 0.25, 95% CI: 0.10–0.63), based on multivariate models adjusted for behavioral, clinical, and nutritional covariates. In the same line, a study identified a significant inverse association for one of the dietary clusters (Vegetable & Fruit) under EWGSOP2 criteria (OR = 0.40, 95% CI: 0.17–0.94) [51], although the remaining three patterns showed no significant associations. Lastly, a cohort study found no significant associations between sarcopenia and any of the dietary patterns assessed (Low Red Meat or Traditional British vs. Low Butter) [50].

Overall, all studies, except one [52], reported adjustment variables including physical activity, smoking, energy intake, and comorbidities or use of medication. Only two studies accounted for variables related to socioeconomic status [44,51], and four studies considered BMI [43,44,48,51].

#### 3.2.3. Methodological Quality of Included Studies

Out of eleven studies, eight were classified as high quality [43,44,46,47,48,49,51,53], while three had methodological limitations related to confounder adjustment, statistical reporting, and outcome assessment [45,50,52]. The quality assessment details are summarized in Table 3.

### 3.3. Meta-Analysis

#### 3.3.1. A Priori and a Posteriori Healthy and Unhealthy Dietary Patterns and Sarcopenia Risk

**Figure 2:**
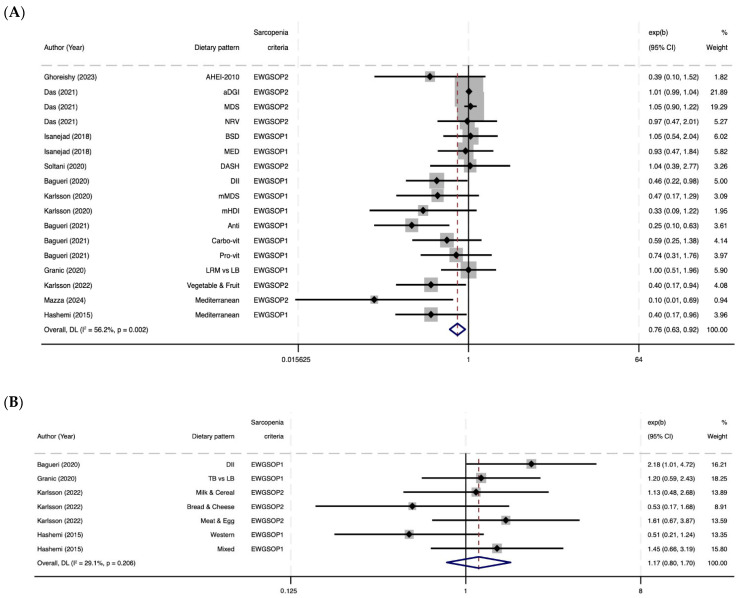
Forest plots of meta-analyses evaluating the association between dietary patterns and sarcopenia risk [43,44,45,46,47,48,49,50,51,52,53]. (**A**). Healthy dietary patterns and sarcopenia risk. (**B**). Unhealthy dietary patterns and sarcopenia risk. AHEI-2010, Alternative Healthy Eating Index 2010; aDGI, Australian Dietary Guideline Index; Anti, anti-inflammatory pattern; BSD, Baltic Sea Diets; Carbo-vit, Carbohydrate–vitamin pattern; DASH, Dietary Approaches to Stop Hypertension; DII, Dietary Inflammatory Index; EWGSOP, European Working Group on Sarcopenia in Older People; MED, Mediterranean Diet Score (Nordic-food-adapted version); MDS, Mediterranean Diet Score; mHDI, Modified Healthy Diet Index; mMDS, Modified Mediterranean Diet Score; LRM vs. LB, Low Red Meat vs. Low Butter; NRV, Nutrient Risk Variable; TB vs. LB: Traditional British vs. Low Butter; Pro-vit, Protein Vitamin pattern. In Figure 2, the DII has been converted for standardization purposes, whereas in Figure 2B, it is presented in its original form, as reported in the primary study.

Figure 2 shows the results of the meta-analysis on the association between dietary patterns and sarcopenia risk. Figure 2A shows that adherence to healthy dietary patterns (n = 11 studies) was significantly associated with a 24% reduced risk of sarcopenia (OR = 0.76, 95% CI: 0.63–0.92). When the analysis was restricted to studies using EWGSOP1 criteria, this association remained statistically significant (n = 10 studies; OR = 0.72, 95% CI: 0.57–0.91; Supplementary Figure S1A). Heterogeneity analysis was moderate among the studies, with I^2^ values of 56.2% (*p* = 0.00) and 52% (*p* = 0.0), respectively.

The meta-regression did not identify any significant moderators in the overall analysis. This includes the EWGSOP diagnostic criteria and its components, such as muscle mass measurement methods (BIA vs. DXA) (*p* = 0.33), different cutoff points for HGS (*p* = 0.94), and physical performance tests including gait speed, the Timed Up and Go, and the Chair Stand Test (*p* = 0.30). Also, no significant associations were found with dietary pattern type (*p* = 0.89), dietary assessment method (*p* = 0.74), study quality (*p* = 0.37), geographic region (*p* = 0.64), variables considered in the adjustment of the multivariate model (e.g., BMI: *p* = 0.37), sex (*p* = 0.64), or age (*p* = 0.76).

In contrast, the analysis between unhealthy dietary patterns and sarcopenia (Figure 2B) showed no significant association (n = 4 studies), OR= 1.17, 95% CI: 0.80–1.70). The meta-analysis restricted to EWGSOP1 (Supplementary Figure S1B) showed similar results (OR = 1.04, 95% CI: 0.66–1.63).

Two additional meta-analyses between a priori and a posteriori healthy dietary patterns and sarcopenia were conducted stratifying by study design and study quality (Supplementary Figures S2 and S3). The analysis based on cross-sectional studies only (excluding three cohort studies with baseline cross-sectional data) and healthy dietary patterns showed a borderline statistically significant inverse association (n = 8 studies; OR = 0.82, 95% CI: 0.67–1.0). When the meta-analysis was restricted to high-quality studies (Supplementary Figure S3), a significant association was maintained (n = 8 studies; OR = 0.73, 95% CI: 0.59–0.91). These results support that low-quality studies did not influence the overall findings.

#### 3.3.2. A Priori Healthy Dietary Patterns and Sarcopenia

**Figure 3:**
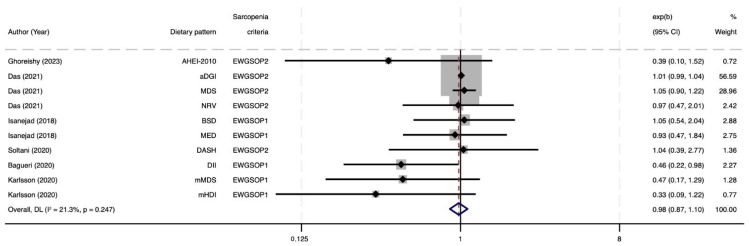
Forest plot of the meta-analysis evaluating the association between a priori healthy dietary patterns and sarcopenia [43,44,45,46,47,48]. AHEI-2010, Alternative Healthy Eating Index 2010; aDGI, Australian Dietary Guideline Index; BSD; Baltic Sea Diets; DASH, Dietary Approaches to Stop Hypertension; DII, Dietary Inflammatory Index; EWGSOP, European Working Group on Sarcopenia in Older People; MED, Mediterranean Diet Score (Nordic-food-adapted version); MDS, Mediterranean Diet Score; mHDI, Modified Healthy Diet Index; mMDS, Modified Mediterranean Diet Score; NRV, Nutrient Risk Variable.

Figure 3 presents a meta-analysis evaluating the association between ten a priori healthy dietary indices (n = 6 studies) and the presence of sarcopenia, using both EWGSOP1 and EWGSOP2 diagnostic criteria. The pooled analysis did not reveal a statistically significant association (OR = 0.98, 95% CI: 0.87–1.10; I^2^ = 21.3%) but a trend towards an inverse association. When exclusively considering studies applying the EWGSOP1 criteria (Supplementary Figure S4A), the results remained non-significant and similar (OR = 0.78, 95% CI: 0.61–1.01; I^2^ = 45.1%).

#### 3.3.3. A Priori and a Posteriori Mediterranean Diet and Risk of Sarcopenia

**Figure 4:**
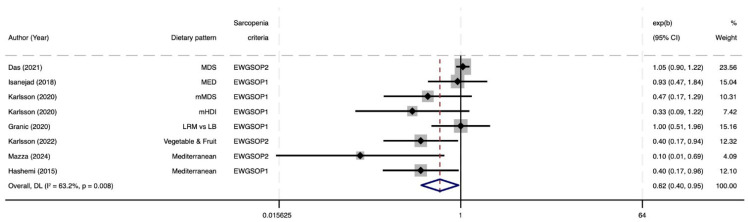
Forest plot of the meta-analysis evaluating the association between Mediterranean dietary patterns and risk of sarcopenia [44,45,48,50,51,52,53]. EWGSOP, European Working Group on Sarcopenia in Older People; LRM vs. LB, Low Red Meat vs. Low Butter; MED, Mediterranean Diet Score (Nordic-food-adapted version); MDS, Mediterranean Diet Score; mHDI, Modified Healthy Diet Index; mMDS, Modified Mediterranean Diet Score.

Figure 4 shows forest plots on the meta-analysis examining the association between eight MD-type dietary patterns (n = 7 studies) and the risk of sarcopenia. Both a priori and a posteriori MD dietary patterns were considered in these analyses, all featuring a low red meat intake; therefore, a dietary pattern characterized by meat restriction was added [39]. The pooled OR indicated a statistically significant 38% reduction in sarcopenia risk among individuals adhering to MD or similar healthy dietary patterns (OR = 0.62, 95% CI: 0.40–0.95). The heterogeneity was moderate (I^2^ = 63.2%), suggesting some variability among study results but not to an extent that undermines the overall finding. In addition, meta-regression analyses did not identify any source of heterogeneity. The meta-analysis restricted to EWGSOP1 (Supplementary Figure S4B) showed similar results (OR = 0.69, 95% CI: 0.50–0.95; I^2^ = 8.7%), with less heterogeneity.

## 4. Publication Bias

Publication bias was assessed using Egger’s test. The intercept was statistically significant (bias = −1.18; *p* = 0.001), indicating the presence of small-study effects. This suggests that studies with larger effect sizes may have been more likely to be published. The slope was not statistically significant (*p* = 0.063). These results are consistent with the observed asymmetry in the funnel plot (Supplementary Figure S5).

## 5. Discussion

The present meta-analysis included eleven studies assessing the association between dietary patterns and sarcopenia risk, the latter defined by the EWGSOP1 and EWGSOP2 criteria enhancing methodological consistency and cross-study comparability, an aspect often overlooked in previous reviews [32,33,55,56]. Furthermore, this study combined results of studies assessing this association considering both a priori and a posteriori dietary patterns, distinguishing further healthy and unhealthy patterns. The healthy and unhealthy dietary pattern classification is widely used in public health and epidemiological research [39,40] and aids comparability but oversimplifies diverse eating practices. As our analysis focused on EWGSOP-based studies mainly from Western contexts, cross-cultural effects are less relevant; however, definitions may not be universally applicable since some foods deemed harmful in one context may hold different cultural or nutritional significance in others [57,58].

Our findings highlight the protective role of healthy dietary patterns in reducing the risk of sarcopenia in older adults. Overall, individuals adhering to healthy dietary patterns (derived a priori or a posteriori) showed a 24% (95% CI: 0.63–0.93) lower risk of sarcopenia with the strongest effect observed for MD patterns (OR = 0.62, 95% CI: 0.40–0.95). Notably, these results are particularly relevant for aging populations, where early nutritional interventions based on healthy dietary patterns could play a key role in preventing or delaying sarcopenia and its associated outcomes.

To our knowledge, this is the first meta-analysis to explore the association between healthy dietary patterns and sarcopenia risk using EWGSOP criteria. A prior meta-analysis on this topic reported no significant association (Pooled OR = 0.95, 95% CI: 0.85–1.06), likely due to limited statistical power (only three studies were included) and potential methodological heterogeneity between the studies. In fact, the three studies used different diagnostic criteria for the assessment of sarcopenia (e.g., EWGSOP1 and Asian Working Group for Sarcopenia) [33]. Similarly, the systematic review conducted by the 2020 Dietary Guidelines Advisory Committee [32] also found inconclusive results. The incongruences found were attributed to differing dietary pattern definitions, methods for dietary and sarcopenia assessment, small sample sizes with few sarcopenia cases, and insufficient adjustment for confounding factors. In contrast, our study examined this association among studies assessing sarcopenia under the same diagnostic criteria to minimize the misclassification of this condition. It is important to acknowledge that all included studies based the definition of sarcopenia on the diagnostic framework of this consensus; no study accounted for molecular or biochemical markers related to sarcopenia, such as IGF-1, pro-inflammatory cytokines, or mitochondrial function indicators, these being makers of disease severity or progression [59,60].

Also, the robustness of our findings was confirmed through sensitivity and subgroup analyses. For instance, when the analysis was restricted to high-quality studies (JBI score ≥ 6; n = 8), the association remained significant, showing a 27% reduction in sarcopenia odds. Likewise, when focusing solely on studies using the EWGSOP1 criteria, a similar association was observed. Finally, meta-regression analyses did not identify any significant sources of heterogeneity in our analyses, including diagnostic criteria and related components, dietary pattern type, dietary assessment method, study quality, study design, geographic region, sex, BMI, or age. This consistency reinforces the validity of our findings and supports the robustness of the protective effect of healthy dietary patterns against sarcopenia across diverse populations and methodological settings.

Healthy dietary patterns were characterized by a high intake of foods known to be beneficial for overall health. These patterns typically include greater consumption of fruits, vegetables, whole grains, unsaturated fats, nuts, legumes, and low-fat dairy products [33]. A recent longitudinal study with up to 30 years of follow-up involving 105,015 participants from two US cohorts (66% women; mean age  =  53 years) demonstrated that higher adherence to healthy dietary patterns was associated with greater odds of achieving healthy aging. Specifically, the OR of healthy ageing for the highest vs. the lowest adherence quintiles ranged from 1.45 (95% CI: 1.35–1.57) for the healthful plant-based diet to 1.86 (95% CI: 1.71–2.01) for the AHEI [61]. Beyond their general health benefits, these patterns also contribute to muscle health. They provide a favorable combination of macro- and micronutrients embedded within a complex food matrix, increasingly recognized for its relevance to musculoskeletal function. More specifically, these diets deliver high amounts of myoprotective nutrients and bioactive compounds, such as antioxidants, polyphenols, and high-quality protein [62]. The synergistic action of these components may help preserve or improve both the quantity and quality of muscle fibers, potentially counteracting key pathophysiological mechanisms underlying sarcopenia [25,62]. A longitudinal study conducted in China among individuals aged 80 and above also indicated that adherence to healthy dietary patterns from midlife into older age is associated with a reduced risk of sarcopenia. This study in particular assessed sarcopenia via the SARC-Calf, though not the EWGSOP2 criteria, yet it highlights the importance of maintaining healthy dietary habits throughout life to reduce the risk of sarcopenia in old age [63].

In particular, the MD, one of the most widely studied and health-promoting plant-based dietary patterns, is characterized by a nutrient-dense composition that may support muscle health through a variety of foods [31,64]. It emphasizes on extra virgin olive oil as the main fat source, a higher intake of fish and seafood providing omega-3 fatty acids, and a unique richness in polyphenols such as hydroxytyrosol, resveratrol, and key micronutrients such as calcium, vitamin D, and B vitamins [65]. Other healthy dietary patterns such as the AHEI-2010, DASH, DGI, and BSD also emphasize the consumption of plant-based foods, healthy fats, and minimally processed ingredients, contributing to a wide array of bioactive compounds, too [43,44,45,46,49]. Together, these dietary patterns limit pro-inflammatory components (e.g., processed meats, sodium, added sugars) and provide high-quality proteins, long-chain omega-3 fatty acids, antioxidant vitamins, fiber, and minerals with anti-inflammatory properties, such as magnesium and selenium. The overlap of nutrients across these patterns complicates the attribution of effects to single foods, but their composite quality appears relevant to muscle maintenance [29,62,66]. Several of these nutrients are proposed to modulate muscle protein synthesis, reduce oxidative stress, and attenuate low-grade systemic inflammation, pathways recognized in the pathogenesis of sarcopenia, particularly in the context of anabolic resistance, mitochondrial dysfunction, and neuromuscular junction degradation [60,67,68]. At the nutrient level, the role of high-quality protein, particularly leucine and other branched-chain amino acids, in stimulating muscle protein synthesis through the activation of mTORC1 while suppressing AMPK signaling is well supported by mechanistic and clinical evidence [25,69]. Plant-based proteins, despite their lower anabolic quality, may also be relevant according to emerging data suggesting that strategic combinations with animal proteins can enhance their efficacy [70]. Evidence for vitamin D and omega-3 fatty acids is mixed, reflecting heterogeneity in study populations, dosage, and endpoints [71,72,73]. Finally, polyphenols abundant in plant-based diets have been shown to suppress NF-κB activation, reduce oxidative stress through Nrf2 pathways, downregulate FOXO3 and atrogenes such as MuRF-1 and atrogin-1, and activate SIRT1 to support mitochondrial biogenesis and function, thereby offering a biologically plausible link between polyphenol-rich dietary patterns (e.g., the MD) and muscle preservation in older adults [74,75].

It is important to emphasize that, when the meta-analysis was restricted to studies evaluating a priori dietary patterns AHEI-2010 [42], aDGI [44], BSD [45], DASH [46], DII [47], mHDI, mMDS [48], MDS, and NRV [44], the overall association with sarcopenia lost statistical significance. A key contributor to these results was the study by Das et al. [44], given the sample size (n = 1705 individuals). This study examined three dietary patterns (aDGI, MDS, and NRV) and their association with sarcopenia risk, which found no significant associations. This inconsistency may be partly explained by variability in the operational definitions of sarcopenia (e.g., differing cut-off values) and heterogeneity in dietary assessment methods across studies. Nevertheless, previous systematic reviews have reported limited and inconclusive evidence regarding the relationship between a priori dietary indices and sarcopenia. For instance, Bloom et al. [76] identified only two eligible studies and highlighted substantial heterogeneity in both exposure definitions (e.g., measures of diet quality indices) and outcomes (e.g., muscle mass, strength, physical performance, or sarcopenia diagnosis). Also, Van Elswyk et al. [33] reviewed three studies that assessed adherence to healthy dietary patterns through a priori approaches, and the pooled findings did not support a significant association with sarcopenia risk (OR = 0.95; 95% CI: 0.85–1.06).

Similarly, unhealthy dietary patterns were not significantly associated with sarcopenia risk. The pooled analysis revealed no significant association (OR = 1.17, 95% CI: 0.80–1.70), which may be partly explained by the limited number of studies included (n = 4), and their limited sample size (n = 1607 individuals). Among the patterns analyzed, the DII [47], Meat & Egg [51], Mixed [53] and Traditional British [50] showed the strongest associations with increased sarcopenia risk. These patterns were characterized by high intake of animal proteins, butter, potato dishes, sweets, and desserts. So far, the relationship between a Westernized or unhealthy dietary pattern and sarcopenia risk remains inconclusive. The most explored unhealthy dietary pattern in relation to this condition is the DII. A recent meta-analysis that combined the results of eleven studies, of which only one study complied with the inclusion criteria of our meta-analysis [47], reported a positive association between the DII and sarcopenia risk, whereby each one-point increase in DII was found to be associated with a 22% higher risk [77]. In our meta-analysis, the Iranian study observed that individuals with the highest DII scores were 2.18 times more likely to have sarcopenia compared to those with the lowest scores [47]. This positive association was further supported by other studies not included in the above. For example, another Chinese cross-sectional study also concluded that higher DII scores were associated with increased risk of muscle strength loss in older adults [78]. Overall, these findings suggest that adopting an anti-inflammatory diet may help reduce sarcopenia risk in older populations.

Among the healthy dietary patterns analyzed, the MD patterns, whether derived a priori or a posteriori—showed the strongest and most consistent protective association with sarcopenia. Indeed, individuals adhering to MD patterns had a 38% lower risk of sarcopenia, which underscores its potential role as a dietary strategy for preserving muscle health in older adults. As aforementioned, the MD is rich in fruits, vegetables, whole grains, fish, and extra virgin olive oil, providing high-quality protein, omega-3 fatty acids, antioxidants, and anti-inflammatory compounds such as polyphenols and dietary fiber. As also previously noted, these components may support muscle health by reducing systemic inflammation, modulating gut microbiota, and lowering pro-inflammatory cytokine production, thereby potentially reducing the risk of sarcopenia [56,79,80,81]. A previous systematic review evaluated whether adherence to the MD is linked to sarcopenia risk; this review did not report a summary estimate since it included only three small studies of mixed populations. The authors concluded that while MD adherence was generally associated with improvements in muscle mass and physical function, the results were less consistent regarding its effects on muscle strength [55]. To the best of our knowledge, our meta-analysis is the first to assess comprehensively the association between MD adherence and sarcopenia risk. By combining results of both a priori and a posteriori definition of the MD, our results support that MD prevents the development of sarcopenia in aging. While a priori- and a posteriori-derived dietary patterns have distinct characteristics, they reflect the overall dietary habits of a group of individuals. In fact, variability in their definition did not affect our results. Their combination in the meta-analysis (four of each) made it possible to assess the association with sarcopenia risk for high vs. low adherence categories of adherence to the MD.

The MD has traditionally been associated with populations residing in Mediterranean regions. However, since its recognition for protective health effects, the MD has been adapted across different regions and assessed using a variety of tools. As an example, our meta-analyses included studies assessing the MD in Australia [44], Iran [53], Finland [45], and the UK [50]. However, despite the varying populations, no heterogeneity was detected for this variable in our results. To measure adherence to the MD, various a priori indices/scores have been developed. For example, there were 22 indices by 2015 that differed in their scoring systems and composition (e.g., Mediterranean Diet Scale: 9 components, range 0–9; Mediterranean Lifestyle Index: 28 components, range 0–28) [82]. These diverse approaches have introduced considerable variability in the food components and scoring criteria used to define adherence to this dietary pattern [62]. In our meta-analysis, we included four a priori MD scores: the MDS; a modified version where the ratio of monounsaturated to saturated fats replaced olive oil intake [44]; the MED score (Nordic-food-adapted version) [45]; and the mMDS, adapted to the Swedish diet [48]. Additionally, as aforementioned, four a posteriori dietary patterns resembling this dietary pattern were included, previously defined as Low Red Meat, Vegetable & Fruit pattern, and the Mediterranean pattern [50,51,52,53]. Two studies contributed most strongly to the overall effect in our meta-analysis [48,52], despite both studies having relatively small sample sizes (n = 170 and n = 254) and one being classified as low quality, potentially inflating its effect size due to bias. Moreover, the association remained significant when the analysis was restricted to studies using EWGSOP1 criteria and diet quality studies. Notably, the exclusion of the low-quality study resulted in a reduction in heterogeneity (from I^2^ = 63.2% to 8.7%), reinforcing the robustness of the observed effect. Further evidence from a cross-sectional study of 2963 participants of the Longevity Check-up 7^+^ project (mean age 72.8; 54% women), which used the Medi-Lite score but a probable assessment of sarcopenia (defined by low muscle strength), and did not meet our inclusion criteria, revealed that high adherence to the MD vs. low adherence was associated with lower odds of probable sarcopenia (OR = 0.60; 95% CI: 0.44–0.81) [83]. Interestingly, some previous studies explored individual and joint effects of the MD and physical activity on sarcopenia prevention defined by EWGSOP2. In one of these studies, aerobic training and MD adherence were assessed in 491 individuals with sarcopenia. The results showed that any of the two were associated with sarcopenia risk or its components [84]. In contrast, in a recently published study conducted within the Toledo Study of Healthy Ageing (n = 1457; mean age 74; 57% women), it was reported that both physical activity and MD adherence were independently associated with a lower sarcopenia prevalence [33]—in particular, with regard to the MD assessed through the MEDAS (Mediterranean Diet Adherence Screener) a 28% reduced risk (95% CI: 0.74–0.91) of sarcopenia defined by EWGSOP2 criteria. Also, this study reported that low adherence to the MD leads to low muscle strength, low skeletal muscle mass, and impaired mobility [33]. Thus, findings of this study agree with those reported in our meta-analysis. Moreover, the studies included in our meta-analysis on the association between MD adherence and sarcopenia risk relied on estimates regardless of physical activity since all studies adjusted for this variable in the analyses.

A notable strength of our meta-analysis is the inclusion of both EWGSOP1 and EWGSOP2 criteria, enabling a multidimensional evaluation of sarcopenia that incorporates both structural (muscle mass) and functional (strength and performance) components. By conducting sensitivity analyses that separated results by diagnostic criteria, including a subgroup analysis restricted exclusively to EWGSOP1-defined studies, we assured the robustness of our findings. Another distinctive aspect of this meta-analysis is the consideration of a priori and a posteriori dietary patterns to increase the statistical power. A priori patterns, such as the MDS, DASH, and AHEI-2010, are structured around established dietary guidelines that promote nutrient-dense and anti-inflammatory foods. In contrast, a posteriori patterns derived through data-driven methods, such as the Western and Mixed patterns [53], may reflect regional or cultural dietary habits that do not align with conventional dietary recommendations. Both types of dietary patterns can be combined to derive pooled estimates, as shown in other meta-analysis [33,85]. Another strength of this study is the approach to study selection: data extraction and quality assessment followed guidance for best practice in systematic reviews, and findings are reported according to the PRISMA guidance.

This study has several limitations that should be acknowledged. First, all the included studies were observational, which limits the ability to establish causal relationships between dietary patterns and sarcopenia risk. Second, errors inherent to dietary assessment cannot be ruled out. Also, common limitations in dietary pattern analyses (lack of standardized food group definitions, varying scoring systems for similar dietary patterns) should be considered [86]. In line with the above, there was variability in how dietary patterns were defined and assessed across the included studies as well as differences in dietary assessment tools (e.g., FFQ, food record). An additional relevant source of variability among studies was the set of covariates included in the adjusted models. However, these variables did not introduce heterogeneity in our analyses. Moreover, we included the same population sample in some cases (e.g., the SARIR cohort from Iran) to consider all possible dietary patterns in order to provide more robust results on the effects of healthy and unhealthy dietary patterns on sarcopenia risk. Finally, potential publication bias cannot be ruled out, as suggested by Egger’s test (*p* = 0.001) and funnel plot asymmetry, indicating that studies reporting stronger associations may have been more likely to be published.

## 6. Conclusions

In summary, this systematic review and meta-analysis supports that healthy dietary patterns, especially those aligned with the MD, could be useful to prevent sarcopenia in adults aged >50 years, as they represent a promising nutritional strategy for sarcopenia prevention. While the evidence for unhealthy dietary patterns remains inconclusive, the observed link between pro-inflammatory diets and increased sarcopenia risk reinforces the relevance of overall diet quality. Future longitudinal and interventional studies, with well-defined and uniformly applied criteria of sarcopenia and definitions of the MD and other dietary patterns, as well as studies incorporating molecular and biochemical markers of the disease, are warranted to disentangle the role of diet in sarcopenia, to ensure comparability and reproducibility across the studies, and to guide the development of dietary guidelines for sarcopenia prevention.

## Figures and Tables

**Table 1:** Characteristics of the studies and the dietary patterns.

Author (Ref)	Country	Study Name	n, % Sex, Age in Years (mean)	Dietary Intake Assessment Method	Dietary Pattern Type	Dietary Pattern Name
Ghoreishy et al. [43]	Iran	SARIR Protocol	300, 50% women, ≥55 (66.8)	117-item FFQ	A priori	AHEI-2010
Das et al. [44]	Australia	CHAMP	1705, 100% men, ≥70 (81)	Diet history questionnaire	A priori	Australian DGI, MDS, NRV
Isanejad et al. [45]	Finland	OSPTRE-EPS	3432, 100% women, ≥65 (67.8)	3-day food record	A priori	BSD, MED Score
Soltani et al. [46]	Iran	SARIR Protocol	300, 50% women, ≥55 (66.8)	117-item FFQ	A priori	DASH diet score
Bagueri et al. [47]	Iran	SARIR Protocol	300, 50% women, ≥55 (66.8)	117-item FFQ	A priori	DII
Karlsson et al. [48]	Sweden	UPSALA	254, 100% men ≥60, (70.9)	7-day diet record	A priori	mHDI, mMDS
Bagueri et al. [49]	Iran	SARIR Protocol	300, 50% women, ≥55 (66.8)	117-item FFQ	A posteriori	Anti, Carbo-vit, Pro-vit
Granic et al. [50]	UK	Newcastle 85+ Study	757, 61% women, ≥85 (85)	24 h MPR	A posteriori	Low Red Meat, Traditional British, Low Butter
Karlsson et al. [51]	Sweden	UPSALA	257, 100% men, ≥60 (70.9)	7-day dietary record	A posteriori	Milk & Cereal, Vegetable & Fruit, Bread & Cheese, Meat & Egg
Mazza et al. [52]	Italy	NR	568, 62% women, ≥50 (61)	FFQ, 24 h recall	A posteriori	Mediterranean
Hashemi et al. [53]	Iran	SARIR Protocol	296, 50% women, ≥55 (66.8)	117-item FFQ	A posteriori	Mediterranean, Western, Mixed

AHEI-2010, Alternative Healthy Eating Index 2010; aDGI, Australian DGI; Anti, anti-inflammatory dietary pattern; BSD, Baltic Sea Diet; CHAMP, The Concord Health and Ageing in Men Project; DII, Dietary Inflammatory Index; FFQ, Food Frequency Questionnaire; MED, Mediterranean Diet Score (Nordic-food-adapted version); MDS, Mediterranean Diet Score; mHDI, Modified Healthy Diet Indicator; mMDS, Modified Mediterranean Diet Score; MPR, Multiple Pass Recall; NRV, Nutrient Risk Variable; OSPTRE-FPS: Osteoporosis Risk Factor and Prevention-Fracture Prevention Study; SARIR: Sarcopenia and its determinants among Iranian elderly; UPPSALA, Uppsala Longitudinal Study of Adult Men.

**Table 2:** Sarcopenia, prevalence, diagnosis criteria, and association with dietary patterns.

		Sarcopenia Components and Cutoff Points				
Author	Method (% Sarcopenia)	Muscle Mass	Muscle Strength	Physical Performance	Results	Adjusted Variables
Ghoreishy et al. [43]	EWGSOP1 (NR)	DXA ASM < 5.45 kg/m^2^ (W) ASM < 7.26 kg/m^2^ (M)	HGS [54]	GS (4 m) <0.8 m/s	AHEI-2010 OR T3 vs. T1: 0.55 (95% CI: 0.22–1.37)	Age, BMI, sex, energy intake, physical activity, smoking, alcohol, medications.
	EWGSOP2 (10.3)	DXA ASM < 5.45 kg/m^2^ (W) ASM < 7.26 kg/m^2^ (M)			AHEI-2010 OR T3 vs. T1: 0.39 (95% CI: 0.10–1.51)	
Das et al. [44]	EWGSOP1 (12.9)	DXA ASM < 7.25 kg/m^2^ (M)	HGS <30 kg	GS (6 m) <0.8 m/s	aDGI OR 1.00 (95% CI: 0.97–1.03) MDS OR 0.55 (95% CI: 0.28–1.09) NRV OR 1.44 (95% CI: 0.72–2.87)	Age, BMI, energy intake, alcohol, physical activity, smoking, MMSE score, marital status, living arrangement, income, SRH, meal service, able to shop for groceries, meal preparation, co-morbidities.
	EWGSOP2 (19.6)	DXA ASM < 7.0 kg/m^2^ (M)	HGS <27 kg		aDGI OR 1.01 (95% CI: 0.98–1.03) MDS OR 1.05 (95% CI: 0.90–1.22) NRV OR 0.97 (95% CI: 0.47–2.01)	
Isanejad et al. [45]	EWGSOP1 (23.2)	DXA RSMI Lowest quartile (M)	HGS Lowest quartile	GS (10 m) Lowest quartile	BSD OR Q4 vs. Q1 1.05 (95% CI: 0.44–1.66) MED Score OR Q4 vs. Q1 0.93 (95% CI: 0.38–1.48)	Age, energy intake, physical activity, smoking, hormone therapy, osteoporosis, rheumatoid arthritis, coronary heart disease, fat mass percentage and income.
Soltani et al. [46]	EWGSOP1 (NR)	DXA ASM < 5.45 kg/m^2^ (W) ASM < 7.26 kg/m^2^ (M)	HGS <20 kg (W) <30 kg (M)	GS (4 m) <0.8 m/s	DASH OR T3 vs. T1 0.78 (95% CI: 0.36–1.67)	Age, sex, energy intake, physical activity, smoking, alcohol, medication.
	EWGSOP2 (10.3)	ASM < 5.5 kg/m^2^ (W) ASM < 7.0 kg/m^2^ (M)			DASH OR T3 vs. T1 1.04 (95% CI: 0.39–2.75)	
Bagueri et al. [47]	EWGSOP1 (17.6)	DXA ASM < 5.45 kg/m^2^ (W) ASM < 7.26 kg/m^2^ (M)	HGS [54]	GS (4 m) <0.8 m/s	DII OR T3 vs. T1 2.18 (95% CI: 1.01–4.74)	Age, sex, energy intake, physical activity, smoking, alcohol, medications, disease history.
Karlsson et al. [48]	EWGSOP1 (21)	DXA ASM < 7.26 kg/m^2^ (M)	HGS <30 kg	GS (4–10 m) <0.8 m/s	mHDI OR T3 vs. T1 0.47 (95% CI: 0.17–1.28) mMDS OR T3 vs. T1 0.33 (95% CI: 0.09–1.23)	Age, BMI, protein intake, physical activity, smoking, inflammation, morbidity, hospital stay, education, living alone.
Bagueri et al. [49]	EWGSOP1 (20)	DXA ASM < 5.45 kg/m^2^ (W) ASM < 7.26 kg/m^2^ (M)	HGS <20 kg (W) <30 kg (M)	GS (4 m) <0.8 m/s	Anti OR T3 vs. T1 0.25 (95% CI: 0.10–0.63) Carbo-vit OR T3 vs. T1 0.59 (95% CI: 0.25–1.36) Pro-vit OR T3 vs. T1 0.74 (95% CI: 0.31–1.76)	Age, sex, energy intake, physical activity, smoking, alcohol, medications, disease history.
Granic et al. [50]	EWGSOP1 (19.2)	BIA SMI < 8.87 kg/m^2^ (M) SMI < 6.67 kg/m^2^ (W)	HGS <16 kg (W) <26 kg (M)	TUG <0.8 m/s	Low Red Meat vs. Low Butter OR 1.00 (95% CI: 0.51–1.96) Traditional British vs. Low Butter OR 1.20 (95% CI: 0.59–2.42) Low Butter (reference)	Age, sex, energy intake, physical activity, smoking, alcohol, medications.
Karlsson et al. [51]	EWGSOP1 (21)	DXA ASM < 7.26 kg/m^2^ (M)	HGS <30 kg	GS (4–10 m) <0.8 m/s	Milk & Cereal: OR T3 vs. T1 0.60 (95% CI: 0.26–1.40) Vegetable & Fruit: OR T3 vs. T1 1.05 (95% CI: 0.45–2.43) Bread & Cheese: OR T3 vs. T1 0.44 (95% CI: 0.14–1.35) Meat & Egg: OR T3 vs. T1 1.72 (95% CI: 0.74–4.02)	Age, BMI, energy intake, physical activity, education, smoking, morbidity.
	EWGSOP2 (19)	DXA ASM < 7.0 kg/m^2^ (M)	HGS <27 kg	GS or 5x chair stand (4–10 m) >15 s or GS < 0.8 m/s	Milk & Cereal: OR T3 vs. T1 1.13 (95% CI: 0.48–2.70 Vegetable & Fruit: OR T3 vs. T1 0.40 (95% CI: 0.17–0.94) Bread & Cheese: OR T3 vs. T1 0.53 (95% CI: 0.17–1.70) Meat & Egg: OR T3 vs. T1 1.61 (95% CI: 0.67–3.87)	
Mazza et al. [52]	EWGSOP2 (9)	BIA ASM < 15 kg (W) ASM < 20 kg (M)	HGS <16 kg (W) <27 kg (M)	NR	Mediterranean OR T3 vs. T1: 0.10 (95% CI: 0.015–0.71)	NR
Hashemi et al. [53]	EWGSOP1 (18.1)	DXA ASM < 5.45 kg/m^2^ (W) ASM < 7.26 kg/m^2^ (M)	HGS [54]	GS (4 m) <0.8 m/s	Mediterranean OR T3 vs. T1 0.40 (95% CI: 0.17–0.97) Western OR T3 vs. T1 0.51 (95% CI: 0.21–1.24) Mixed OR T3 vs. T1 1.45 (95% CI: 0.66–3.19)	Age, sex, energy intake, physical activity, smoking, alcohol, medications, disease history.

AHEI-2010, Alternative Healthy Eating Index 2010; aDGI, Dietary Guideline Index; Anti, anti-inflammatory dietary pattern; ASM, Appendicular skeletal muscle mass; BSD, Baltic Sea diets; BIA, Bioelectrical impedance analysis; BMI, Body mass index; Carbo-vit, Carbohydrate-vitamin dietary pattern; DASH, Dietary Approaches to Stop Hypertension; DII, Dietary Inflammatory Index; DXA, Dual-energy X-ray Absorptiometry; EWGSOP, European Working Group on Sarcopenia in Older People; GS, Gait speed; HGS, Handgrip strength; MED, Mediterranean Diet Score (Nordic-food-adapted version); MDS, Mediterranean Diet Score; MHDI, Modified Healthy Diet Index; mMDS, Modified Mediterranean Diet Score; NR, not reported; NRV, Nutrient Risk Variable; RSMI, relative skeletal muscle index; SMI, skeletal muscle index; Pro-vit, protein vitamin pattern; SRH, self-rated health; TUG, Timed Up and Go test; W, women; M, men.

**Table 3:** Methodological quality of cross-sectional studies (JBI checklist).

Study	Q1	Q2	Q3	Q4	Q5	Q6	Q7	Q8	Total Score	Quality
Ghoreishy et al. [43]									8	High
Das et al. [44]	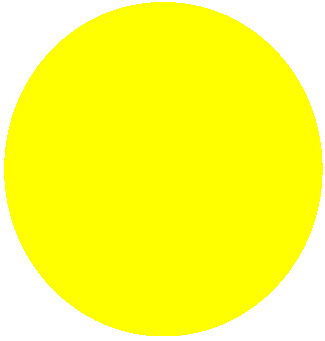				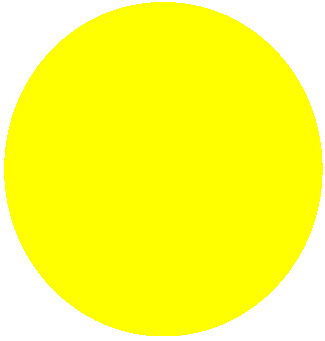				6	High
Isanejad et al. [45]	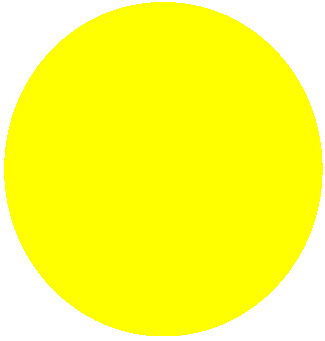	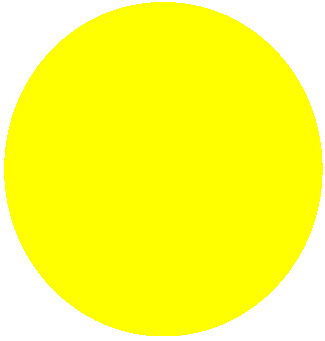			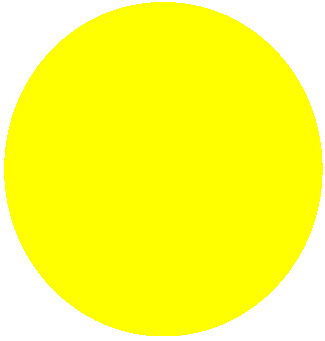		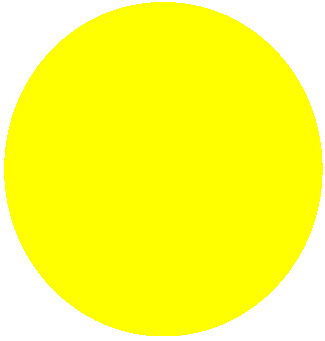		4	Low
Soltani et al. [46]									8	High
Bagueri et al. [47]							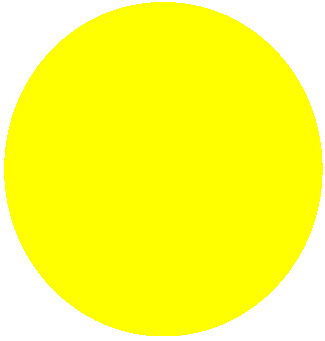		7	High
Karlsson et al. [48]								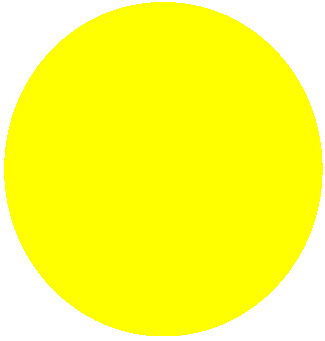	7	High
Bagueri et al. [49]							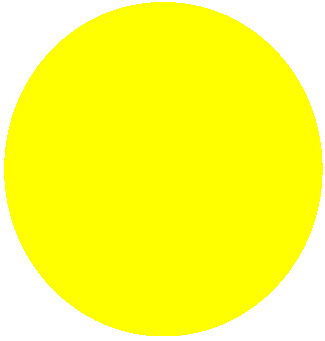		7	High
Granic et al. [50]						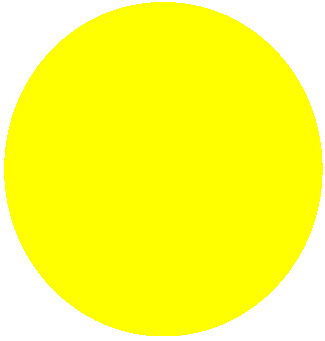	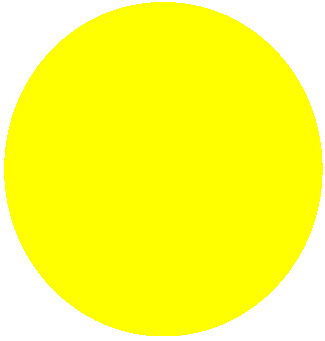	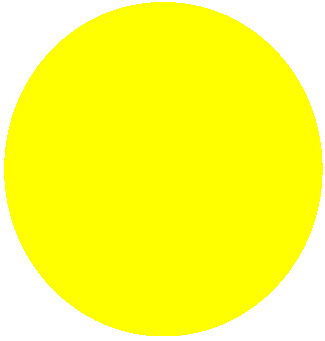	5	Low
Karlsson et al. [51]								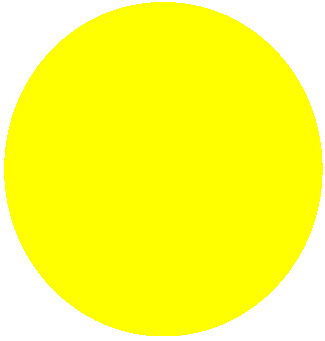	7	High
Mazza et al. [52]				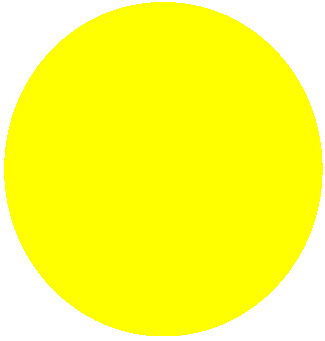		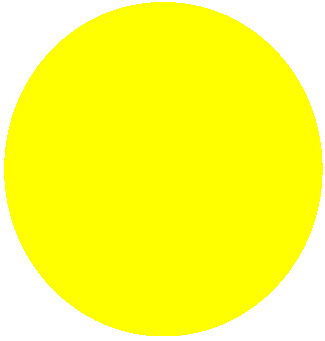		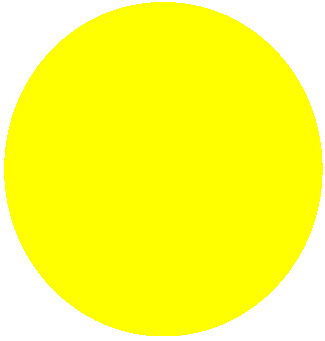	5	Low
Hashemi et al. [53]									8	High

Scoring was based on the JBI Critical Appraisal Checklists (Yes = 1, No/Unclear = 0). Q1. Were the criteria for inclusion in the sample clearly defined?; Q2. Were the study subjects and the setting described in detail?; Q3. Was the exposure measured in a valid and reliable way?; Q4. Were objective, standard criteria used for measurement of the condition?; Q5. Were confounding factors identified?; Q6. Were strategies to deal with confounding factors stated?; Q7. Were the outcomes measured in a valid and reliable way?; Q8. Was appropriate statistical analysis used? Color coding was applied for clarity in quality assessment: green indicates a fulfilled criterion (score = 1), yellow indicates a non-fulfilled criterion (score = 0). In the Total Score column, green denotes high methodological quality (≥6 points), whereas red denotes low quality (<6 points).

## Data Availability

Data from published articles.

## Supplementary Materials

The following supporting information can be downloaded at: https://www.mdpi.com/article/10.3390/nu17172764/s1, Supplementary Table S1: PRISMA 2020 checklist; Supplementary Table S2: Characteristics of healthy and unhealthy dietary patterns; Supplementary Figure S1: Forest plot of the meta-analysis evaluating the association between a priori and a posteriori dietary patterns and risk of sarcopenia, restricted to EWGSOP1; Supplementary Figure S2: Forest plot of the meta-analysis evaluating the association between healthy dietary patterns a priori and a posteriori and sarcopenia by cross-sectional studies; Supplementary Figure S3: Forest plot of the meta-analysis evaluating the association between a priori and a posteriori dietary patterns and sarcopenia in high-quality studies; Supplementary Figure S4: Forest plot of the meta-analysis evaluating the association between a priori and a posteriori dietary patterns and sarcopenia, restricted to EWGSOP1; Supplementary Figure S5: Funnel plot of healthy dietary patterns and sarcopenia according to EWGSOP1 and EWGSOP.

## Abbreviations

The following abbreviations are used in this manuscript:

AHEI-2010	Alternative Healthy Eating Index 2010
aDGI	Australian Dietary Guideline Index
Anti	Anti-inflammatory dietary pattern
ASM	Appendicular Skeletal Muscle Mass
BSD	Baltic Sea Diet
Bread & Cheese	Bread & Cheese dietary pattern
BIA	Bioelectrical Impedance Analysis
BMI	Body Mass Index
CHAMP	Concord Health and Ageing in Men Project
Carbo-vit	Carbohydrate–Vitamin dietary pattern
DASH	Dietary Approaches to Stop Hypertension
DII	Dietary Inflammatory Index
DXA	Dual-energy X-ray Absorptiometry
EWGSOP1	European Working Group on Sarcopenia in Older People 1
EWGSOP2	European Working Group on Sarcopenia in Older People 2
FFQ	Food Frequency Questionnaire
GS	Gait Speed
HGS	Handgrip Strength
JBI	The Joanna Briggs Institute checklist
LRM	Low Red Meat
LB	Low Butter
MD	Mediterranean diet
MDS	Mediterranean Diet Score
MED	Mediterranean Diet Score (Nordic-food-adapted version)
MEDAS	Mediterranean Diet Adherence Screener
mHDI	Modified Healthy Diet Index
Meat & Egg	Meat & Egg dietary pattern
Milk & Cereal	Milk & Cereal dietary pattern
mMDS	Modified Mediterranean Diet Score
MPR	Multiple Pass Recall
NRV	Nutrient Risk Variable
OSPTRE-FPS	Osteoporosis Risk Factor and Prevention-Fracture Prevention Study
PRISMA	Preferred Reporting Items for Systematic Reviews and Meta-Analyses
Pro-vit	Protein Vitamin pattern
PROSPERO	Prospective Register of Systematic Reviews
RSMI	Relative Skeletal Muscle Index
SARIR	Sarcopenia and its determinants among Iranian elderly
SMI	Skeletal Muscle Index
TUG	Timed Up and Go test
Traditional British	Traditional British dietary pattern
UPPSALA	Uppsala Longitudinal Study of Adult Men
Vegetable & Fruit	Vegetable & Fruit dietary pattern
Western	Western dietary pattern

## References

[1] Yuan S., Larsson S.C. (2023). Epidemiology of sarcopenia: Prevalence, risk factors, and consequences. Metabolism.

[2] Cruz-Jentoft A.J., Bahat G., Bauer J., Boirie Y., Bruyère O., Cederholm T., Cooper C., Landi F., Rolland Y., Sayer A.A. (2019). Sarcopenia: Revised European consensus on definition and diagnosis. Age Ageing.

[3] Arnal-Gómez A., Cebrià IIranzo M.A., Tomas J.M., Tortosa-Chuliá M.A., Balasch-Bernat M., Sentandreu-Mañó T., Forcano S., Cezón-Serrano N. (2021). Using the updated EWGSOP2 definition in diagnosing sarcopenia in Spanish older adults: Clinical approach. J. Clin. Med..

[4] Van Ancum J.M., Alcazar J., Meskers C.G.M., Nielsen B.R., Suetta C., Maier A.B. (2020). Impact of using the updated EWGSOP2 definition in diagnosing sarcopenia: A clinical perspective. Arch. Gerontol. Geriatr..

[5] Chiu W.C., Kao T.W., Peng T.C. (2025). Prevalence of sarcopenia in Asian older adults: A comparison of nine diagnostic criteria across different regions. Exp. Gerontol..

[6] Cruz-Jentoft A.J., Baeyens J.P., Bauer J.M., Boirie Y., Cederholm T., Landi F., Martin F.C., Michel J.P., Rolland Y., Schneider S.M. (2010). Sarcopenia: European consensus on definition and diagnosis: Report of the European Working Group on Sarcopenia in Older People. Age Ageing.

[7] Beaudart C., Alcazar J., Aprahamian I., Batsis J.A., Yamada Y., Prado C.M., Reginster J.Y., Sanchez-Rodriguez D., Lim W.S., Sim M. (2025). Health outcomes of sarcopenia: A consensus report by the outcome working group of the Global Leadership Initiative in Sarcopenia (GLIS). Aging Clin. Exp. Res..

[8] Kirk B., Cawthon P.M., Arai H., Ávila-Funes J.A., Barazzoni R., Bhasin S., Binder E.F., Bruyere O., Cederholm T., Chen L.K. (2024). The conceptual definition of sarcopenia: Delphi consensus from the Global Leadership Initiative in Sarcopenia (GLIS). Age Ageing.

[9] Chen L.K., Woo J., Assantachai P., Auyeung T.W., Chou M.Y., Iijima K., Jang H.C., Kang L., Kim M., Kim S. (2020). Asian Working Group for Sarcopenia: 2019 consensus update on sarcopenia diagnosis and treatment. J. Am. Med. Dir. Assoc..

[10] Meng S., He X., Fu X., Zhang X., Tong M., Li W., Zhang W., Shi X., Liu K. (2024). The prevalence of sarcopenia and risk factors in older adults in China: A systematic review and meta-analysis. Front. Public Health.

[11] Reiss J., Iglseder B., Alzner R., Mayr-Pirker B., Pirich C., Kässmann H., Kreutzer M., Dovjak P., Reiter R. (2019). Consequences of applying the new EWGSOP2 guideline instead of the former EWGSOP guideline for sarcopenia case finding in older patients. Age Ageing.

[12] Petermann-Rocha F., Balntzi V., Gray S.R., Lara J., Ho F.K., Pell J.P., Celis-Morales C. (2022). Global prevalence of sarcopenia and severe sarcopenia: A systematic review and meta-analysis. J. Cachexia Sarcopenia Muscle.

[13] Stuck A.K., Tsai L.T., Freystaetter G., Vellas B., Kanis J.A., Rizzoli R., Kressig K.S., Armbrecht G., Da Silva J.A.P., Dawson-Hughes B. (2023). Comparing prevalence of sarcopenia using twelve sarcopenia definitions in a large multinational European population of community-dwelling older adults. J. Nutr. Health Aging.

[14] Miao Y., Xie L., Song J., Cai X., Yang J., Ma X., Chen S., Xie P. (2024). Unraveling the causes of sarcopenia: Roles of neuromuscular junction impairment and mitochondrial dysfunction. Physiol. Rep..

[15] Kim M.J., Sinam I.S., Siddique Z., Jeon J.H., Lee I.K. (2023). The link between mitochondrial dysfunction and sarcopenia: An update focusing on the role of pyruvate dehydrogenase kinase 4. Diabetes Metab. J..

[16] Priego T., Martín A.I., González-Hedström D., Granado M., López-Calderón A., Cardalini D. (2021). Role of hormones in sarcopenia. Vitamins and Hormones.

[17] Sung J.Y., Lee M.J., Kim J. (2025). Relationship between lifestyle and physical fitness among older women with sarcopenia. Int. J. Mol. Sci..

[18] Calvani R., Picca A., Coelho-Júnior H.J., Tosato M., Marzetti E., Landi F. (2023). Diet for the prevention and management of sarcopenia. Metabolism.

[19] Ely I.A., Phillips B.E., Smith K., Wilkinson D.J., Piasecki M., Breen L., Larsen M.S., Atherton P.J. (2023). A focus on leucine in the nutritional regulation of human skeletal muscle metabolism in ageing, exercise and unloading states. Clin. Nutr..

[20] Cailleaux P.E., Déchelotte P., Coëffier M. (2024). Novel dietary strategies to manage sarcopenia. Curr. Opin. Clin. Nutr. Metab. Care.

[21] Kazemi A., Speakman J.R., Soltani S., Djafarian K. (2020). Effect of calorie restriction or protein intake on circulating levels of insulin-like growth factor I in humans: A systematic review and meta-analysis. Clin. Nutr..

[22] Chen M., Wang Y., Deng S., Lian Z., Yu K. (2022). Skeletal muscle oxidative stress and inflammation in aging: Focus on antioxidant and anti-inflammatory therapy. Front. Cell Dev. Biol..

[23] Cruz-Jentoft A.J., Dawson-Hughes B., Scott D., Sanders K.M., Rizzoli R. (2020). Nutritional strategies for maintaining muscle mass and strength from middle age to later life: A narrative review. Maturitas.

[24] Phillips N., Gray S.R., Combet E., Witard O.C. (2024). Long-chain n-3 polyunsaturated fatty acids for the management of age- and disease-related declines in skeletal muscle mass, strength and physical function. Curr. Opin. Clin. Nutr. Metab. Care.

[25] Robinson S.M., Reginster J.Y., Rizzoli R., Shaw S.C., Kanis J.A., Bautmans I., Bischoff-Ferrari H., Bruyère O., Cesari M., Dawson-Hughes B. (2018). Does nutrition play a role in the prevention and management of sarcopenia?. Clin. Nutr..

[26] Robinson S., Granic A., Cruz-Jentoft A.J., Sayer A.A. (2023). The role of nutrition in the prevention of sarcopenia. Am. J. Clin. Nutr..

[27] Sabir Z., Dierkes J., Hjartåker A., Rosendahl-Riise H. (2023). The association of dietary patterns with muscle mass and strength in old age: The Hordaland Health Study. Eur. J. Nutr..

[28] Wingrove K., Lawrence M.A., McNaughton S.A. (2022). A systematic review of the methods used to assess and report dietary patterns. Front. Nutr..

[29] Davis J.A., Mohebbi M., Collier F., Loughman A., Staudacher H., Shivappa N., Hébert J.R., Pasco J.A., Jacka F.N. (2021). The role of diet quality and dietary patterns in predicting muscle mass and function in men over a 15-year period. Osteoporos. Int..

[30] Silva R., Pizato N., da Mata F., Figueiredo A., Ito M., Pereira M.G. (2018). Mediterranean diet and musculoskeletal-functional outcomes in community-dwelling older people: A systematic review and meta-analysis. J. Nutr. Health Aging.

[31] Álvarez-Bustos A., Coelho-Junior H.J., Carnicero J.A., García-García F.J., Marzetti E., Rodriguez-Mañas L. (2025). Adherence to the Mediterranean diet and physical activity in relation to sarcopenia: A cross-sectional epidemiological cohort study. Aging Clin. Exp. Res..

[32] Boushey C., Ard J., Bazzano L., Heymsfield S., Mayer-Davis E., Sabaté J., Snetselaar L., Van Horn L., Schneeman B., English L.K. (2020). Dietary Patterns and Sarcopenia: A Systematic Review.

[33] Van Elswyk M.E., Teo L., Lau C.S., Shanahan C.J. (2022). Dietary patterns and the risk of sarcopenia: A systematic review and meta-analysis. Curr. Dev. Nutr..

[34] Peña-Ordóñez G.G., Bustamante-Montes L.P., Ramírez-Duran N., Sánchez-Castellano C., Cruz-Jentoft A.J. (2017). Populations and outcome measures used in ongoing research in sarcopenia. Aging Clin. Exp. Res..

[35] Ramadas A., Law H.H., Krishnamoorthy R., Ku J.W.S., Mohanty P., Lim M.Z.C., Shyam S. (2022). Diet quality and measures of sarcopenia in developing economies: A systematic review. Nutrients.

[36] Carvalho do Nascimento P.R., Bilodeau M., Poitras S. (2021). How do we define and measure sarcopenia? A meta-analysis of observational studies. Age Ageing.

[37] Page M.J., McKenzie J.E., Bossuyt P.M., Boutron I., Hoffmann T.C., Mulrow C.D., Shamseer L., Tetzlaff J.M., Akl E.A., Brennan S.E. (2021). The PRISMA 2020 statement: An updated guideline for reporting systematic reviews. BMJ.

[38] Munn Z., Moola S., Lisy K., Riitano D., Tufanaru C. (2015). Methodological guidance for systematic reviews of observational epidemiological studies reporting prevalence and cumulative incidence data. Int. J. Evid. Based Healthc..

[39] World Health Organization (2020). Healthy Diet.

[40] Food and Agriculture Organization of the United Nations, World Health Organization (2019). Sustainable Healthy Diets: Guiding Principles.

[41] Schwingshackl L., Schwedhelm C., Hoffmann G., Lampousi A.M., Knüppel S., Iqbal K., Bechthold A., Schlesinger S., Boeing H. (2017). Food groups and risk of all-cause mortality: A systematic review and meta-analysis of prospective studies. Am. J. Clin. Nutr..

[42] Shivappa N., Steck S.E., Hurley T.G., Hussey J.R., Hébert J.R. (2014). Designing and developing a literature-derived, population-based dietary inflammatory index. Public. Health Nutr..

[43] Ghoreishy S.M., Koujan S.E., Hashemi R., Heshmat R., Motlagh A.D., Esmaillzadeh A. (2023). Relationship between healthy eating index and sarcopenia in elderly people. BMC Geriatr..

[44] Das A., Cumming R.G., Naganathan V., Blyth F., Le Couteur D.G., Handelsman D.J., Waite L.M., Ribeiro R.V., Simpson S.J., Hirani V. (2021). Associations between nutrient intakes and dietary patterns with different sarcopenia definitions in older Australian men: The Concord Health and Ageing in Men Project. Public. Health Nutr..

[45] Isanejad M., Sirola J., Mursu J., Rikkonen T., Kröger H., Tuppurainen M., Erkkilä A.T. (2018). Association of the Baltic Sea and Mediterranean diets with indices of sarcopenia in elderly women: The OSTPRE-FPS study. Eur. J. Nutr..

[46] Soltani S., Hashemi R., Heshmat R., Motlagh A.D., Esmaillzadeh A. (2020). Association of dietary approaches to stop hypertension eating style and risk of sarcopenia. Sci. Rep..

[47] Bagheri A., Soltani S., Hashemi R., Heshmat R., Motlagh A.D., Esmaillzadeh A. (2020). Inflammatory potential of the diet and risk of sarcopenia and its components. Nutr. J..

[48] Karlsson M., Becker W., Michaëlsson K., Cederholm T., Sjögren P. (2020). Associations between dietary patterns at age 71 and the prevalence of sarcopenia 16 years later. Clin. Nutr..

[49] Bagheri A., Hashemi R., Heshmat R., Motlagh A.D., Esmaillzadeh A. (2021). Patterns of nutrient intake in relation to sarcopenia and its components. Front. Nutr..

[50] Granic A., Mendonça N., Sayer A.A., Hill T.R., Davies K., Siervo M., Mathers J.C., Jagger C. (2020). Effects of dietary patterns and low protein intake on sarcopenia risk in the very old: The Newcastle 85+ study. Clin. Nutr..

[51] Karlsson M., Becker W., Cederholm T.E., Byberg L. (2022). A posteriori dietary patterns in 71-year-old Swedish men and the prevalence of sarcopenia 16 years later. Br. J. Nutr..

[52] Mazza E., Ferro Y., Maurotti S., Micale F., Boragina G., Russo R., Lascala L., Sciacqua A., Gazzaruso C., Montalcini T. (2024). Association of dietary patterns with sarcopenia in adults aged 50 years and older. Eur. J. Nutr..

[53] Hashemi R., Motlagh A.D., Heshmat R., Esmaillzadeh A., Payab M., Yousefinia M., Siassi F., Pasalar P., Baygi F. (2015). Diet and its relationship to sarcopenia in community-dwelling Iranian elderly: A cross-sectional study. Nutrition.

[54] Merkies I.S., Schmitz P.I., Samijn J.P., Meché F.G., Toyka K.V., van Doorn P.A. (2000). Assessing grip strength in healthy individuals and patients with immune-mediated polyneuropathies. Muscle Nerve.

[55] Papadopoulou S.K., Detopoulou P., Voulgaridou G., Tsoumana D., Spanoudaki M., Sadikou F., Papadopoulou V.G., Zidrou C., Chatziprodromidou I.P., Giaginis C. (2023). Mediterranean diet and sarcopenia features in apparently healthy adults over 65 years: A systematic review. Nutrients.

[56] Granic A., Sayer A.A., Robinson S.M. (2019). Dietary patterns, skeletal muscle health, and sarcopenia in older adults. Nutrients.

[57] Hu F.B. (2002). Dietary pattern analysis: A new direction in nutritional epidemiology. Curr. Opin. Lipidol..

[58] Imamura F., Micha R., Khatibzadeh S., Fahimi S., Shi P., Powles J., Mozaffarian D., Global Burden of Diseases Nutrition and Chronic Diseases Expert Group (NutriCoDE) (2015). Dietary quality among men and women in 187 countries in 1990 and 2010: A systematic assessment. Lancet Glob. Health.

[59] Sharma B., Dabur R. (2020). Role of pro-inflammatory cytokines in regulation of skeletal muscle metabolism: A systematic review. Curr. Med. Chem..

[60] Jimenez-Gutierrez G.E., Martínez-Gómez L.E., Martínez-Armenta C., Pineda C., Martínez-Nava G.A., Lopez-Reyes A. (2022). Molecular mechanisms of inflammation in sarcopenia: Diagnosis and therapeutic update. Cells.

[61] Tessier A.J., Wang F., Korat A.A., Eliassen A.H., Chavarro J., Grodstein F., Li J., Liang L., Willett W.C., Sun Q. (2025). Optimal dietary patterns for healthy aging. Nat. Med..

[62] Granic A., Sayer A.A., Cooper R., Robinson S.M. (2024). Nutrition in the prevention and treatment of skeletal muscle ageing and sarcopenia: A single nutrient, a whole food, and a whole diet approach. Proc. Nutr. Soc..

[63] Bai A., Xu W., Liang Y., Jiang Y., Lin Z. (2023). Dietary patterns from mid-through later-life in relation to sarcopenia risk over 20 years among Chinese community-dwelling oldest old individuals. Clin. Nutr..

[64] Trajkovska Petkoska A., Ognenoska V., Trajkovska-Broach A. (2025). Mediterranean diet: From ancient traditions to modern science—A sustainable way towards better health, wellness, longevity, and personalized nutrition. Sustainability.

[65] Naureen Z., Dhuli K., Donato K., Aquilanti B., Velluti V., Matera G., Iaconelli A., Bertelli M. (2022). Foods of the Mediterranean diet: Tomato, olives, chili pepper, wheat flour and wheat germ. J. Prev. Med. Hyg..

[66] Yu X., Pu H., Voss M. (2024). Overview of anti-inflammatory diets and their promising effects on non-communicable diseases. Br. J. Nutr..

[67] Moresi V., Renzini A., Cavioli G., Seelaender M., Coletti D., Gigli G., Cedola A. (2022). Functional nutrients to ameliorate neurogenic muscle atrophy. Metabolites.

[68] Prokopidis K., Chambers E., Ni Lochlainn M., Witard O.C. (2021). Mechanisms linking the gut-muscle axis with muscle protein metabolism and anabolic resistance: Implications for older adults at risk of sarcopenia. Front. Physiol..

[69] McColl T.J., Clarke D.C. (2023). Kinetic modeling of leucine-mediated signaling and protein metabolism in human skeletal muscle. iScience.

[70] Carbone J.W., Pasiakos S.M. (2022). The role of dietary plant and animal protein intakes on mitigating sarcopenia risk. Curr. Opin. Clin. Nutr. Metab. Care.

[71] Qi P., Fu X., Zhao D., Li C., Lu Y., Li N. (2024). Effects of vitamin D supplementation on muscle strength in middle-aged and elderly individuals: A retrospective, propensity score-matched study. Front. Nutr..

[72] Bird J.K., Troesch B., Warnke I., Calder P.C. (2021). The effect of long chain omega-3 polyunsaturated fatty acids on muscle mass and function in sarcopenia: A scoping systematic review and meta-analysis. Clin. Nutr. ESPEN.

[73] Dupont J., Dedeyne L., Dalle S., Koppo K., Gielen E. (2019). The role of omega-3 in the prevention and treatment of sarcopenia. Aging Clin. Exp. Res..

[74] Centonze M., Caruso E.A., De Nunzio V., Cofano M., Saponara I., Pinto G., Notarnicola M. (2025). The antiaging potential of dietary plant-based polyphenols: A review on their role in cellular senescence modulation. Nutrients.

[75] Prado C.M., Landi F., Chew S.T.H., Atherton P.J., Molinger J., Ruck T., Gonzalez M.C. (2022). Advances in muscle health and nutrition: A toolkit for healthcare professionals. Clin. Nutr..

[76] Bloom I., Shand C., Cooper C., Robinson S., Baird J. (2018). Diet quality and sarcopenia in older adults: A systematic review. Nutrients.

[77] Diao H., Yan F., He Q., Li M., Zheng Q., Zhu Q., Fang F., Cui W. (2023). Association between dietary inflammatory index and sarcopenia: A meta-analysis. Nutrients.

[78] Pu R., Man Q., Song S., Jia S., Liu Z., Zhang X., Zhang J., Song P. (2025). The dietary inflammatory index and sarcopenia in older adults in four Chinese provinces: A cross-sectional study. Nutrients.

[79] Andreo-López M.C., Contreras-Bolívar V., García-Fontana B., García-Fontana C., Muñoz-Torres M. (2023). The influence of the Mediterranean dietary pattern on osteoporosis and sarcopenia. Nutrients.

[80] Itsiopoulos C., Mayr H.L., Thomas C.J. (2022). The anti-inflammatory effects of a Mediterranean diet: A review. Curr. Opin. Clin. Nutr. Metab. Care.

[81] Dominguez L.J., Veronese N., Smith L., Ragusa F.S., Schirò P., Di Bella G., Barbagallo M. (2025). Associations between adherence to the Mediterranean diet and incident sarcopenia in prospective cohort studies. Nutrients.

[82] Hernández-Ruiz A., García-Villanova B., Guerra-Hernández E.J., Amiano P., Azpiri M., Molina-Montes E. (2015). Description of indexes based on the adherence to the Mediterranean dietary pattern: A review. Nutr. Hosp..

[83] Cacciatore S., Calvani R., Marzetti E., Picca A., Coelho-Júnior H.J., Martone A.M., Massaro C., Tosato M., Landi F. (2023). Low adherence to Mediterranean diet is associated with probable sarcopenia in community-dwelling older adults: Results from the Longevity Check-Up (Lookup) 7+ project. Nutrients.

[84] Coelho-Júnior H.J., Calvani R., Picca A., Cacciatore S., Tosato M., Landi F., Marzetti E. (2023). Combined aerobic training and Mediterranean diet is not associated with a lower prevalence of sarcopenia in Italian older adults. Nutrients.

[85] Hong S.H., Bae Y.J. (2024). Association of dietary vegetable and fruit consumption with sarcopenia: A systematic review and meta-analysis. Nutrients.

[86] Pilleron S., Pérès K., Jutand M.A., Helmer C., Dartigues J.F., Samieri C., Féart C. (2018). Dietary patterns and risk of self-reported activity limitation in older adults from the Three-City Bordeaux Study. Br. J. Nutr..

