# Supplementary material for: Healthy Dietary Patterns and Risk of Sarcopenia in Adults Aged > 50 Years: A Systematic Review and Meta-Analysis Considering EWGSOP1 and EWGSOP2 Criteria
Source: Nutrients. 2025 Aug 26;17(17):2764. doi: 10.3390/nu17172764 (PMC12430432; doi:10.3390/nu17172764)
Supplement: Supplementary file 1 [file nutrients-17-02764-s001.zip › nutrients-3789315-supplementary.pdf]

nutrients

MDPI

Healthy Dietary Patterns and Risk of Sarcopenia in Adults Aged > 50 Years: A Systematic Review and Meta-Analysis Considering EWGSOP1 and EWGSOP2 Criteria

Supplementary Tables & Figures

Supplementary Table S1. PRISMA 2020 checklist **1-4**

Supplementary Table S2. Characteristics of healthy and unhealthy dietary patterns **5,6**

Supplementary Figure S1. Forest plot of the meta-analysis evaluating the association between *a priori* and *a posteriori* dietary patterns and risk of sarcopenia, restricted to EWGSOP1 .... **7**

A. Healthy dietary pattern
B. Unhealthy dietary patterns

Supplementary Figure S2. Forest plot of the meta-analysis evaluating the association between *a priori* and *a posteriori* healthy dietary patterns and sarcopenia in cross-sectional studies **8**

Supplementary Figure S3. Forest plot of the meta-analysis evaluating the association between *a priori* and *a posteriori* dietary patterns and sarcopenia in high-quality studies **9**

Supplementary Figure S4. Forest plot of the meta-analysis evaluating the association between *a priori* and *a posteriori* dietary patterns and sarcopenia, restricted to EWGSOP1 .... **10**

A. *A priori* healthy dietary patterns
B. *A priori* and *a posteriori* Mediterranean dietary patterns

Supplementary Figure S5. Funnel plot of healthy dietary patterns and sarcopenia according to EWGSOP1 and EWGSOP2. .... **11**

Supplementary Table S1. PRISMA 2020 Checklist

Section and Topic	Item #	Checklist item	Location where item is reported
**TITLE**			
Title	1	Identify the report as a systematic review.	P1
**ABSTRACT**			
Abstract	2	See the PRISMA 2020 for Abstracts checklist.	P1
**INTRODUCTION**			
Rationale	3	Describe the rationale for the review in the context of existing knowledge.	P2
Objectives	4	Provide an explicit statement of the objectives or questions the review addresses.	P3
**METHODS**			
Eligibility criteria	5	Specify the inclusion and exclusion criteria for the review and how studies were grouped for the syntheses.	P4,P5
Information sources	6	Specify all databases, registers, websites, organisations, reference lists and other sources searched or consulted to identify studies. Specify the date when each source was last searched or consulted.	P4,P6
Search strategy	7	Present the full search strategies for all databases, registers and websites, including any filters and limits used.	P4,P5
Selection process	8	Specify the methods used to decide whether a study met the inclusion criteria of the review, including how many reviewers screened each record and each report retrieved, whether they worked independently, and if applicable, details of automation tools used in the process.	P4,P5
Data collection process	9	Specify the methods used to collect data from reports, including how many reviewers collected data from each report, whether they worked independently, any processes for obtaining or confirming data from study investigators, and if applicable, details of automation tools used in the process.	P4,P5,P6
Data items	10a	List and define all outcomes for which data were sought. Specify whether all results that were compatible with each outcome domain in each study were sought e.g. for all measures, time points, analyses, and if not, the methods used to decide which results to collect.	P7

	10b	List and define all other variables for which data were sought e.g. participant and intervention characteristics, funding sources. Describe any assumptions made about any missing or unclear information.	P7
Study risk of bias assessment	11	Specify the methods used to assess risk of bias in the included studies, including details of the tools used, how many reviewers assessed each study and whether they worked independently, and if applicable, details of automation tools used in the process.	P5,P6

**Section and Topic**	**Item #**	**Checklist item**	**Location where item is reported**
Effect measures	12	Specify for each outcome the effect measures e.g. risk ratio, mean difference used in the synthesis or presentation of results.	P8
Synthesis methods	13a	Describe the processes used to decide which studies were eligible for each synthesis e.g. tabulating the study intervention characteristics and comparing against the planned groups for each synthesis item #5.	P10-P11
	13b	Describe any methods required to prepare the data for presentation or synthesis, such as handling of missing summary statistics, or data conversions.	P6
	13c	Describe any methods used to tabulate or visually display results of individual studies and syntheses.	P10-P11
	13d	Describe any methods used to synthesize results and provide a rationale for the choices. If meta-analysis was performed, describe the models, methods to identify the presence and extent of statistical heterogeneity, and software packages used.	P6
	13e	Describe any methods used to explore possible causes of heterogeneity among study results e.g. subgroup analysis, metaregression.	P6
	13f	Describe any sensitivity analyses conducted to assess robustness of the synthesized results.	P6
Reporting bias assessment	14	Describe any methods used to assess risk of bias due to missing results in a synthesis arising from reporting biases.	P5-P6
Certainty assessment	15	Describe any methods used to assess certainty or confidence in the body of evidence for an outcome.	P5-P6
**RESULTS**			
Study selection	16a	Describe the results of the search and selection process, from the number of records identified in the search to the number of studies included in the review, ideally using a flow diagram.	P7
	16b	Cite studies that might appear to meet the inclusion criteria, but which were excluded, and explain why they were excluded.	P7
Study characteristics	17	Cite each included study and present its characteristics.	P7-P9

Risk of bias in studies	18	Present assessments of risk of bias for each included study.	P12
Results of individual studies	19	For all outcomes, present, for each study: a summary statistics for each group where appropriate and b an effect estimate and its precision e.g. confidence/credible interval, ideally using structured tables or plots.	P10-P11
Results of syntheses	20a	For each synthesis, briefly summarise the characteristics and risk of bias among contributing studies.	P12
	20b	Present results of all statistical syntheses conducted. If meta-analysis was done, present for each the summary estimate and its precision e.g. confidence/credible interval and measures of statistical heterogeneity. If comparing groups, describe the direction of the effect.	P12-P15
**Section and Topic**	**Item #**	**Checklist item**	**Location where item is reported**
	20c	Present results of all investigations of possible causes of heterogeneity among study results.	P12-P15
	20d	Present results of all sensitivity analyses conducted to assess the robustness of the synthesized results.	P12-P15
Reporting biases	21	Present assessments of risk of bias due to missing results arising from reporting biases for each synthesis assessed.	P12
Certainty of evidence	22	Present assessments of certainty or confidence in the body of evidence for each outcome assessed.	P12-P15
**DISCUSSION**			
Discussion	23a	Provide a general interpretation of the results in the context of other evidence.	P15
	23b	P4	P19
	23c	Discuss any limitations of the review processes used.	P18-P20
	23d	Discuss implications of the results for practice, policy, and future research.	P20
**OTHER INFORMATION**			
Registration and protocol	24a	Provide registration information for the review, including register name and registration number, or state that the review was not registered.	P3
	24b	Indicate where the review protocol can be accessed, or state that a protocol was not prepared.	P3
	24c	Describe and explain any amendments to information provided at registration or in the protocol.	P3
Support	25	Describe sources of financial or non-financial support for the review, and the role of the funders or sponsors in the review.	P20

Competing interests	26	Declare any competing interests of review authors.	P21
Availability of data, code and other materials	27	Report which of the following are publicly available and where they can be found: template data collection forms; data extracted from included studies; data used for all analyses; analytic code; any other materials used in the review.	P2

Supplementary Table S2. Characteristic of healthy and unhealthy dietary patterns

Study	Characteristics of Healthy Dietary Patterns	Characteristics of Unhealthy Dietary Patterns
Ghoreishy et al. [43] *A priori*	**AHEI-2010**<br>High intake of fruits, vegetables, whole grains, nuts, legumes, and healthy fats such as long-chain omega-3 fatty acids (DHA, EPA, ALA); along with low intake of sugar-sweetened beverages and fruit juice, red and processed meats, trans fats, and salt.	—
Das et al. [44] *A priori*	**aDGI**<br>High intake of fruits, vegetables, (total and whole grains), meat and dairy alternatives; and low intake of discretionary foods (fats, salt, sugars, alcohol).<br><br>**MDS**<br>Not reported<br><br>**NRV**<br>High intake of protein, dietary fiber, essential fatty acids (linolenic and linoleic), vitamins (A, C, K, folate, riboflavin), and minerals (magnesium, calcium, iron, zinc).	—
Isanejad et al. [45] *A priori*	**BSD**<br>High intake of apples, pears, berries, root vegetables, legumes, nuts, mushrooms, whole grains, fish, and low-fat milk; with low intake of processed meats and total fat (E%).<br>**MED**<br>High intake of vegetables, legumes, nuts (excluding potatoes), fruits, cereals, and fish; characterized by a healthy fat profile (high PUFA + MUFA: SFA ratio), moderate alcohol consumption, and low intake of meats, sausages, eggs, and dairy products.	—
Soltani et al. [46] *A priori*	**DASH**<br>High intake of fruits, vegetables, legumes, nuts, lowfat dairy, and whole grains; combined with low intake of sodium, red and processed meats, sugar-sweetened beverages, and sweets.	—
Bagueri et al. [47] *A priori*	**DII**<br>High intake of energy-dense foods rich in protein, total fat, and dietary fiber; a wide range of vitamins (A, B1, B2, B3, B6, B12, C, D, E); minerals including iron, zinc, magnesium, and selenium; and bioactive compounds such as β-carotene, caffeine, garlic, tea, onion, and pepper.	—

Karlsson et al. [48] *A priori*	**mHDI**<br>High intake of foods rich in SFA, PUFA, protein, carbohydrates, dietary fiber, fruits and vegetables, cholesterol, and fish; with low intake of added sucrose (E%).<br>**mMDS**<br>High intake of vegetables, fruits, cereals, fish, meats, and dairy products, with moderate alcohol consumption; and a favorable PUFA/SFA ratio.	—
Bagueri et al. [49]<br>*A posteriori*	**Pro-vitamin**<br>High intake of foods rich in protein and saturated fat; essential minerals including calcium, zinc, and selenium; B-complex vitamins such as B2, B9, B5, and B12; as well as fat-soluble vitamins A, D, and K.<br>**Anti-inflammatory**<br>High intake of foods rich in unsaturated fats, including monounsaturated (MUFA) and omega-3 fatty acids; antioxidant nutrients such as vitamin E and vitamin B6; minerals such as copper, magnesium, iron, and sodium, and bioactive compounds like caffeine.<br>**Carbo-vitamin**<br>High intake of foods rich in simple carbohydrates (fructose, glucose), dietary fiber, water-soluble vitamins including biotin, thiamine (B1), and vitamin C, as well as potassium, and the trace element chromium.	—
Granic et al.<br>[50]<br>*A posteriori*	**Low Red Meat**<br>High intake of fish/seafood and low intake of legumes, sweets, and soups.	**Traditional British**<br>High intake of red meats, potatoes, legumes, vegetables, and desserts.<br>**Low Butter**<br>High intake of potatoes, vegetables, and soup.
Karlsson et al.<br>[51]<br>*A posteriori*	**Vegetable & Fruit**<br>High intake of vegetables, green salad, fruit, poultry, rice, and pasta.	**Milk & Cereal**<br>High intake of milk, and cereals.<br>**Bread & Cheese**<br>High intake of bread, cheese, jam, and added sugars.<br>**Meat & Egg**<br>High intake of meat, eggs and potatoes, and low intake of fermented milk.
Mazza et al.<br>[52]<br>*A posteriori*	**Mediterranean**<br>High intake of legumes, cereals, fruits, vegetables, and low intake of meat, fish, and eggs.	
Hashemi et al.<br>[53]<br>*A posteriori*	**Mediterranean**<br>High intake of olive oil, carotenoid-rich vegetables, tomatoes, whole grains, nuts, fish, fresh and dried fruits, and pickles.	**Western**<br>High intake of tea, soy, sweets, and fast food.<br>**Mixed**<br>High intake of animal protein, legumes, potatoes, and refined grains.

ALA, alpha-linolenic acid; AHEI-2010, Alternative Healthy Eating Index 2010; aDGI, Australian Dietary Guideline Index; BDS, Baltic Diet Score; DASH, Dietary Approaches to Stop Hypertension; DHA, docosahexaenoic acid; DII, Dietary Inflammatory Index; E%, percentage energy, EPA, eicosapentaenoic acid; EWGSOP, European Working Group on Sarcopenia in Older People; MED, Mediterranean Diet Score; mHDI,

Modified Healthy Diet Index; mMDS, Modified Mediterranean Diet Score; MUFA, monounsaturated fatty acids; NRV, Nutrient Risk Variable; PUFA, polyunsaturated fatty acids; SFA, saturated fatty acids.

A

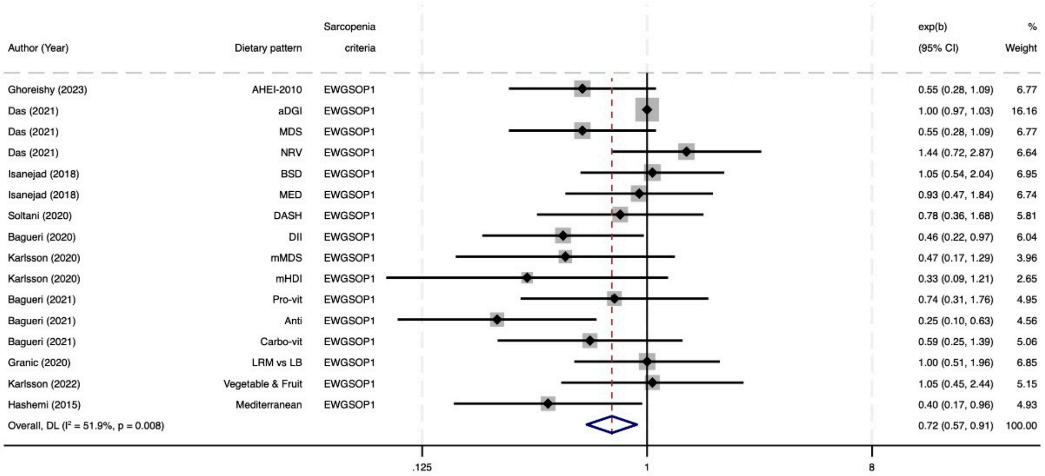

B

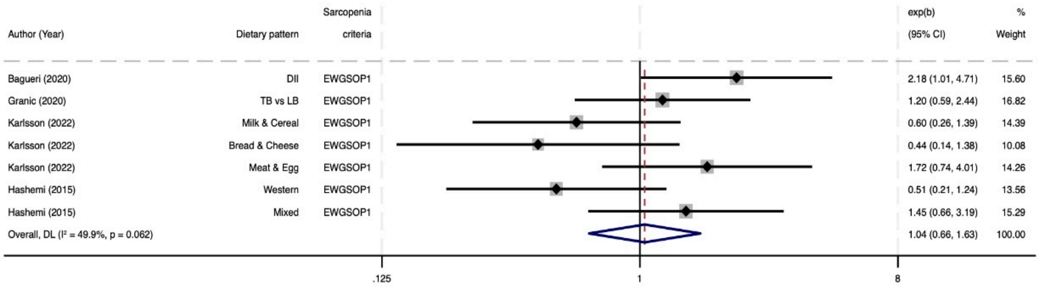

Supplementary Figure S1. Forest plot of the meta-analysis evaluating the association between *a priori* and *a posteriori* dietary patterns and risk of sarcopenia, restricted to EWGSOP1. **A.** Healthy dietary patterns and risk of sarcopenia **B.** Unhealthy dietary patterns and the risk of sarcopenia. AHEI-2010, Alternative Healthy Eating Index 2010; aDGI, Australian Dietary Guideline Index; Anti, Anti-inflammatory pattern; BSD, Baltic Sea Diet; Carbo-vit, Carbohydrate–vitamin pattern; DASH, Dietary Approaches to Stop Hypertension; DII, Dietary Inflammatory Index; EWGSOP, European Working Group on Sarcopenia in Older People; MED, Mediterranean Diet Score (Nordic food–adapted version); MED, Mediterranean Diet Score; mHDI, Modified Healthy Diet Index; mMDS, Modified Mediterranean Diet Score; LRM vs LB, Low Red Meat vs Low Butter; NRV, Nutrient Risk Variable; TB vs LB, Traditional British vs Low Butter; Pro-vitamin, Protein–Vitamin pattern.

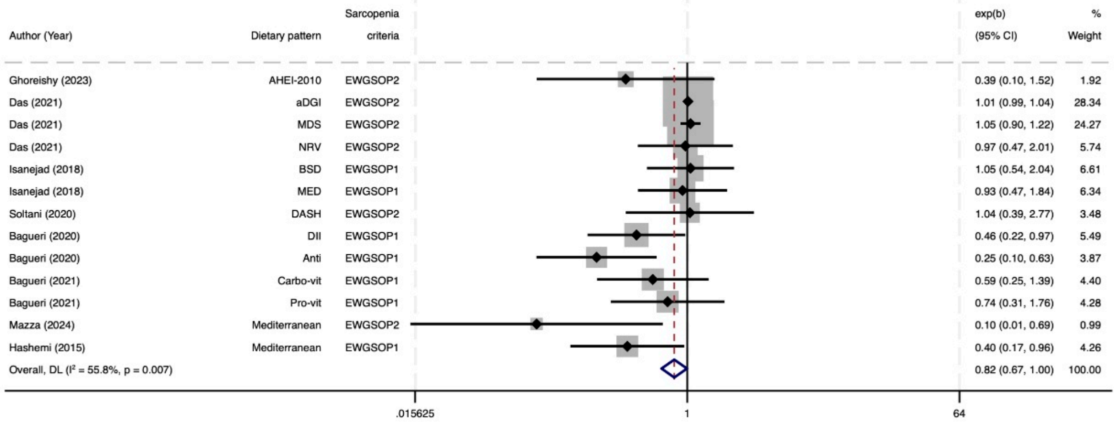

Supplementary Figure S2. Forest plot of the meta-analysis evaluating the association between *a priori* and *a posteriori* healthy dietary patterns and sarcopenia in cross-sectional studies**.** AHEI, Alternative Healthy Eating Index; aDGI, Australian Dietary Guideline Index; Anti, Anti-inflammatory pattern; BSD, Baltic Sea Diet; Carbo-vit, Carbohydrate–vitamin pattern; DASH, Dietary Approaches to Stop Hypertension; DII, Dietary Inflammatory Index; EWGSOP, European Working Group on Sarcopenia in Older People; MED, Mediterranean Diet Score (Nordic food–adapted version); MED, Mediterranean Diet Score; mHDI, Modified Healthy Diet Index; mMDS, Modified Mediterranean Diet Score; NRV, Nutrient Risk Variable; Pro-vit, Protein–vitamin pattern.

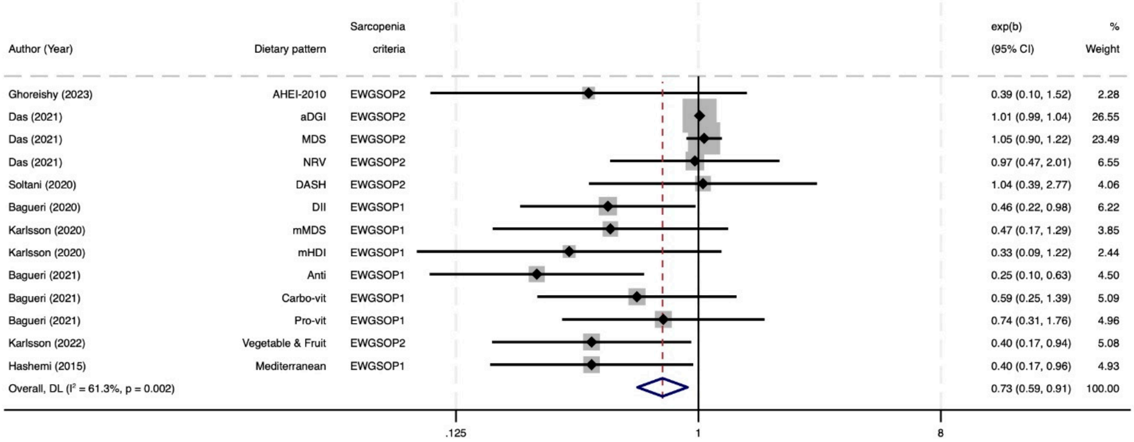

Supplementary Figure S3. Forest plot of the meta-analysis evaluating the association between studies of *a priori* and *a posteriori* dietary patterns and sarcopenia by high quality studies. AHEI, Alternative Healthy Eating Index; aDGI, Australian Dietary Guideline Index; Anti, Anti-inflammatory pattern; BSD, Baltic Sea Diet; Carbo-vit, Carbohydrate–vitamin pattern; DASH, Dietary Approaches to Stop Hypertension; DII, Dietary Inflammatory Index; EWGSOP, European Working Group on Sarcopenia in Older People; MED, Mediterranean Diet Score (Nordic food–adapted version); MED, Mediterranean Diet Score; mHDI, Modified Healthy Diet Index; mMDS, Modified Mediterranean Diet Score; NRV, Nutrient Risk Variable; Pro-vit, Protein–vitamin pattern.

A

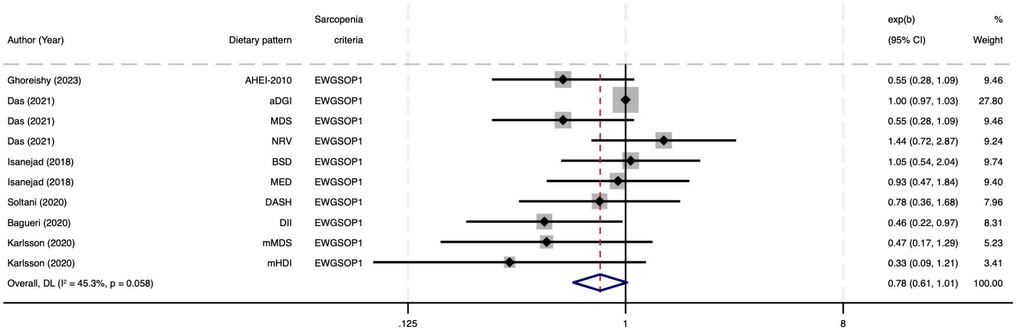

B

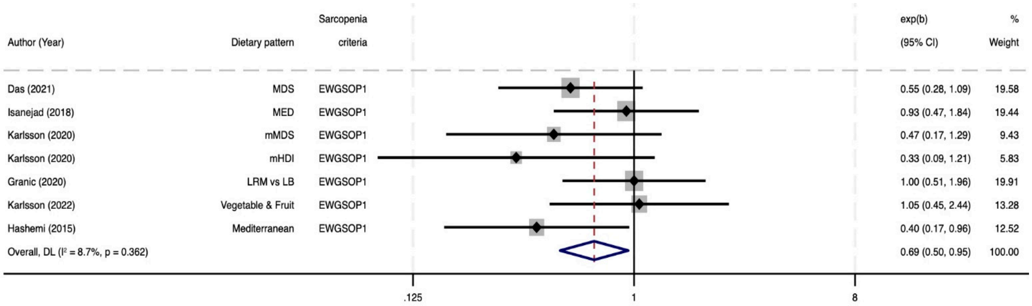

Supplementary Figure S4. Forest plot of the meta-analysis evaluating the association between *a priori* and *a posteriori* dietary patterns and sarcopenia, restricted to EWGSOP1. **A.** *A priori* healthy dietary patterns **B**. A *priori* and *a posteriori* mediterranean dietary patterns. AHEI, Alternative Healthy Eating Index; aDGI, Australian Dietary Guideline Index; BSD, Baltic Sea Diet; DASH, Dietary Approaches to Stop Hypertension; DII, Dietary Inflammatory Index; EWGSOP, European Working Group on Sarcopenia in Older People; MED, Mediterranean Diet Score (Nordic food–adapted version); MED, Mediterranean Diet Score; mHDI, Modified Healthy Diet Index; mMDS, Modified Mediterranean Diet Score; NRV, Nutrient Risk Variable.

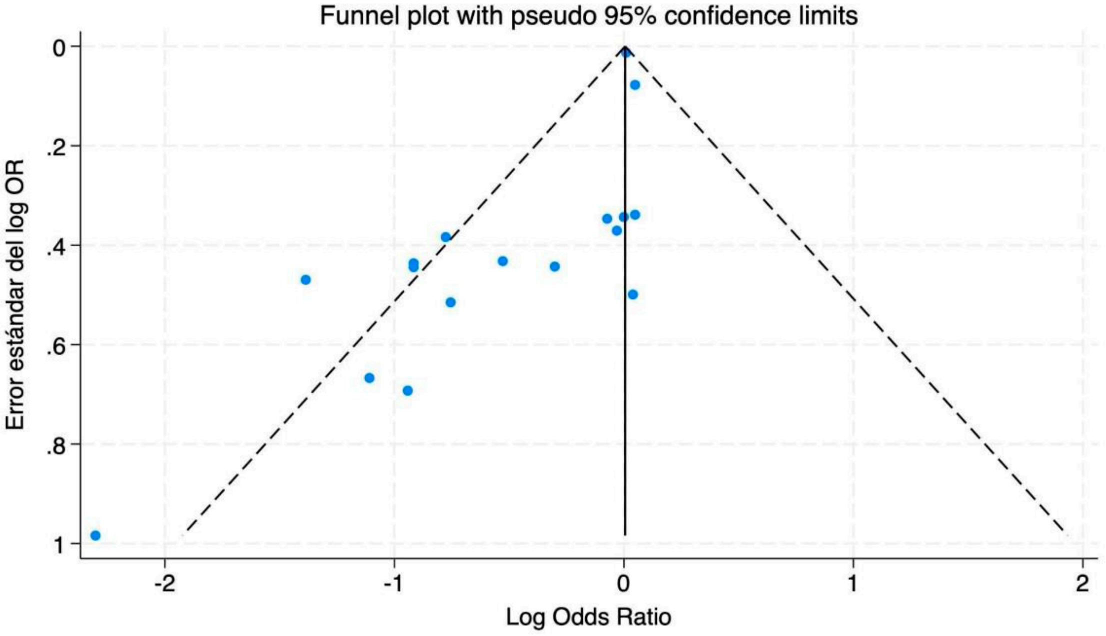

Supplementary Figure S5. Funnel plot of healthy dietary patterns and sarcopenia according to EWGSOP1 and EWGSOP2
